# Unexpected complexity of the Aquaporin gene family in the moss *Physcomitrella patens*

**DOI:** 10.1186/1471-2229-8-45

**Published:** 2008-04-22

**Authors:** Jonas ÅH Danielson, Urban Johanson

**Affiliations:** 1 Department of Biochemistry, Center for Molecular Protein Science, Center for Chemistry and Chemical Engineering, Lund University, PO Box 124, S-221 00 Lund, Sweden

## Abstract

**Background:**

Aquaporins, also called major intrinsic proteins (MIPs), constitute an ancient superfamily of channel proteins that facilitate the transport of water and small solutes across cell membranes. MIPs are found in almost all living organisms and are particularly abundant in plants where they form a divergent group of proteins able to transport a wide selection of substrates.

**Results:**

Analyses of the whole genome of *Physcomitrella patens *resulted in the identification of 23 MIPs, belonging to seven different subfamilies, of which only five have been previously described. Of the newly discovered subfamilies one was only identified in *P. patens *(Hybrid Intrinsic Protein, HIP) whereas the other was found to be present in a wide variety of dicotyledonous plants and forms a major previously unrecognized MIP subfamily (X Intrinsic Proteins, XIPs). Surprisingly also some specific groups within subfamilies present in *Arabidopsis thaliana *and *Zea mays *could be identified in *P. patens*.

**Conclusion:**

Our results suggest an early diversification of MIPs resulting in a large number of subfamilies already in primitive terrestrial plants. During the evolution of higher plants some of these subfamilies were subsequently lost while the remaining subfamilies expanded and in some cases diversified, resulting in the formation of more specialized groups within these subfamilies.

## Background

Water transport across cell membranes is essential for life and in order to facilitate the transport of water and other small polar molecules across hydrophobic membranes, living organisms have evolved a wide array of membrane integral protein channels. These proteins, termed major intrinsic proteins (MIPs), form a large and evolutionarily conserved superfamily of channel proteins, found in all types of organisms, including eubacteria, archaea, fungi, animals and plants [1,2]. MIPs are present in many different tissues in mammals and are likely to be of major importance for many different diseases [reviewed in [3]], either directly or indirectly through their involvement in transport and water balance regulation. This general physiological involvement of MIPs has stimulated a growing interest in the molecular mechanisms responsible for regulation and substrate specificity. In plants the functions of MIPs are more complex and their physiological roles are not as clear [reviewed in [4,5]]. However, the mere number of different MIPs in plants implies their importance, and it is likely that some isoforms play key roles in events such as rapid cell elongation and drought adaptation through their involvement in water transport regulation [6]. In order to fully understand whole plant water relations and the transport of other small polar molecules at a molecular level it is necessary to identify the complete set of MIPs along with their substrate specificities and expression patterns.

A comprehensive phylogenetic study of MIPs [7] supports the classification of two main evolutionary groups. Aquaporins (AQPs) originally thought to specifically transport water, and glycerol-uptake facilitators or aquaglyceroporins (GLPs) facilitating the transport of a variety of small neutral molecules. Although the MIPs form passive channels, the permeability of the membrane is regulated by controlling the amount of different MIPs and also in some cases by phosphorylation/dephosphorylation of the channels. Structures from x-ray and electron crystallography of MIPs [8-14] show a tetrameric quaternary structure in which each monomer consists of six membrane spanning helices (H1 to H6) connected by five loops (A-E). Loop B (cytoplasmic) and loop E (extracellular) form two half-membrane spanning helices (HB and HE) and interact with each other from opposing sides through two highly conserved aspargine-proline-alanine (NPA) boxes, forming a narrow region of the pore. A constriction region about 8 Å from the NPA boxes toward the periplasmic side, termed the aromatic/arginine (ar/R) region, is formed by two residues from H2 and H5 and two residues from loop E. This region forms a primary selection filter and is a major checkpoint for solute permeability [[15], and references therein].

Plant MIPs form a large and divergent superfamily of proteins with more than thirty identified members encoded in each of the genomes of *Arabidopsis thaliana *[16,17], *Zea mays *[18] and *Oryza sativa *[19]. These large numbers of MIPs likely reflect a wide diversity in substrate specificity, localisation, transcriptional and posttranslational regulation. Based on sequence similarity plant MIPs have been divided into five subfamilies; the plasma membrane intrinsic proteins (PIPs), the tonoplast intrinsic proteins (TIPs), the nodulin-26 like intrinsic proteins (NIPs), the small basic intrinsic proteins (SIPs) and the GlpF-like intrinsic protein (GIPs) [7,16,20]. The GIPs have so far only been identified in *Physcomitrella patens *and another closely related moss [20]. Each of the other subfamilies can be further divided into groups based on sequence similarity [16]. Even though all MIPs in higher plants phylogenetically belong to the AQP clade of MIPs [7] they are not all highly specific for water. Several studies have shown plant MIPs to be permeable also to other molecules, for example TIPs have been reported to facilitate urea and ammonia transport [21-23]; NIPs to transport glycerol [24], ammonia [25], lactic acid [26], boron [27] and silicon [28]; PIPs have been postulated to be able to facilitate CO_2 _diffusion [29,30] and for the SIPs water transport has only been reported for the SIP1 subgroup [31]. The difference in transport specificity is likely due to major differences in the ar/R filter of plant MIPs, as has been suggested for MIPs in *A. thaliana, Z. mays *and *O. sativa *[32,33].

*P. patens *is a moss (bryophyte) and as such diverged from the lineage leading to higher plants approximately 443–490 million years ago, before the evolution of vascular plants [34]. This makes *P. patens *a valuable source of information in evolutionary comparisons with higher plants and any common features found can be expected to be present in most terrestrial plants. In addition *P. patens *has properties that make it an attractive plant model for future functional studies, above all the possibility of homologous recombination [information about the use of *P. patens *can be found in two excellent reviews by David Cove [35,36]]. An assembled genome of *P. patens *(*circa *480 Mbp), based on 8.1 times coverage, has recently been released by the Joint Genome Institute [37,38] and has made it possible to extend the analysis of gene family evolution back to basal land plant lineages. Such an analysis has previously been described for the expansin superfamily of proteins [39] and we now present a similar analysis of the MIP superfamily. In agreement with the expansin study, we also hypothesised that *P. patens *were to have a simpler superfamily structure due to less need of cell-specific expression, a hypothesis that was partially proven wrong by the data collected for *P. patens*. In our analysis we did not only identify the five previously defined subfamilies (PIP, TIP, NIP, SIP and GIP) but also found two previously uncategorised MIP subfamilies; the hybrid intrinsic proteins (HIPs) and the uncategorized X intrinsic proteins (XIPs), a subfamily which we found also to be present in many other plant species. This data implies that MIP subfamilies evolved early on in plants and that the existence of diverse subfamilies reflects differences in subcellular localisation, substrate specificity, transcriptional and/or posttranslational regulation already of importance in primitive plants, whereas the specificity needed only in higher plants (e.g. cell specific expression in vascular tissue and seeds) is covered by the MIP groups that evolved later within the subfamilies present in higher plants.

In this study we try to address plant MIP function from an evolutionary perspective by comparing the whole set of MIPs in a primitive land plant (the moss *P. patens*) with those of two higher plants (*A. thaliana *and *Z. mays*). By annotating the whole MIP superfamily in *P. patens *we also lay the foundation for future functional studies in a plant system allowing homologous recombination and all advantages of this, such as knocking out/replacing endogenous genes.

## Results

### Identification of *Physcomitrella patens *MIPs

The recent sequencing of the moss *P. patens *genome [37,38] has for the first time made it possible to identify all MIP genes in a more primitive plant and hence to make conclusions on the molecular evolution of the MIP superfamily of proteins. Searches of the *Physcomitrella patens ssp patens v1.1 *database (PpDB) at JGI, using the 35 protein sequences of the complete set of *A. thaliana *MIPs (AtMIPs) [16], resulted in identification of 23 different genes encoding *P. patens *MIPs (PpMIPs) (Table 1). Two genes were identical at nucleotide level and therefore only one protein sequence (PpPIP2;4), representing both genes, was included in further analyses. PpGIP1;1, a *P. patens *MIP previously described in detail by Gustavsson et al [20] was also included in the PpMIP set which were then reaching a total of 23 full length MIPs. Four genes encoding partial MIP-like sequences were also identified. Of these, three were either partial or contained premature stop codons and therefore considered to be non-functional pseudogenes (pseudoPIP#1, pseudoPIP#2 and pseudoNIP#1). The fourth sequence might represent a functional MIP encoding gene, but was situated in a short contig interrupted by a large sequencing gap after the identified exon and could therefore not be included in the analysis (referred to as partialNIP#1). The JGI gene models were manually inspected and considered correct for most PpMIP genes. However, for some genes a different annotation of the coding sequence in the genomic sequence was favoured either by cDNA sequences or due to a better conservation of subfamily specific sequences and gene structure. These alternative assignations of exons, specified in Table 1, were used in all translations and analyses in this paper.

**Table 1:** Proposed systematic names for all *Physcomitrella patens *MIPs

**New name**^a^	**Borstlap**^b^	**PpDB**^c^	**EST**^d^	**ProteinID**^e^	**Comments**^f^
PIP1;1	-	PIP1	Y	62169	
PIP1;2	PIP1	PIP	Y	166091	
PIP1;3	PIP1	PIP	Y	171662	
PIP2;1	-	PIP	Y	202226	
PIP2;2	PIP2	PIP	Y	209703	
PIP2;3	PIP2	PIP	Y	196472	
PIP2;4	-	PIP	?	135286	Identical to 83986^g^
-	-	PIP	?	83986	Identical to 135286^g^
PIP3;1	-	PIP2	?	68172	
PseudoPIP#1	-	-^h^	?	113412	Pseudogene, PIP-like, based on ProteinID = 113412 but encoding 123 amino acids in two exons
PseudoPIP#2	-	-	?	-	Pseudogene, PIP-like, encoding 83 amino acids in one exon
TIP6;1	-	TIP	Y	73809	
TIP6;2	TIP	TIP	Y	191107	
TIP6;3	TIP	-^h^	Y	214518	
TIP6;4	TIP	TIP	Y	219971	
NIP3;1	-	NIP5	?	94322	The PpDB classification refers to ProteinID = 147365 which is a truncated version
NIP5;1	-	NIP4	Y	115513	Misannotated: delete the first amino acid and add exon 1 (68 amino acids)
NIP5;2	NIP	NIP4	Y	186237	Misannotated: delete first eleven amino acids and add exon 1 (68 amino acids)
NIP5;3		NIP4	Y	179749	Misannotated: delete first seven amino acids and add exon 1 (66 amino acids)
NIP6;1	-	NIP	?	16763	Misannotated: add exon 1 (65 amino acids) and extend last exon 24 amino acids
PartialNIP#1	-	Possibly an aquaporin^i^	?	103774	Possibly a full length gene (NIP5) but the genomic sequence is only 825 bp long and interrupted by a 34 kb gap. The model which the classification refers to (ProteinID = 103774) is completely wrong, but in the opposite direction is an exon encoding 103 amino acids.
PseudoNIP#1	-	-	?	73549	Pseudogene, NIP-like, delete first 22 amino acids from model
SIP1;1	SIP	SIP	?	112053	
SIP1;2	SIP	SIP	Y	200882	
GIP1;1	-	PpGlP1-1	Y	171260	
HIP1;1	-	-^h^	?	91611	Misannotated, we removed 141 aa from beginning of exon 1, 22 aa from end of exon 2 and 15 aa from beginning of exon 3
XIP1;1	-	TIP1	Y	71087	The PpDB classification refers to ProteinID = 26452 which is a truncated version
XIP1;2	-	TIP	Y	71489	Misannotated, removed 15 amino acids from exon 2 and replaced exon 1 (now 31 aa) The PpDB classification refers to ProteinID = 47381 which is a truncated version

^a ^Proposed new names for *P. patens *MIPs. ^b ^Classification used in Borstlap (2002). ^c ^Classification used to describe gene models by Shizong Ma in PpDB. ^d ^Matching ESTs in PpDB: Y = Yes, ? = Not found. ^e ^Protein ID number for the protein or related protein in PpDB. ^f ^Alternative exon/intron positions proposed and used in this paper and odd features of genes and/or proteins encoded. ^g ^both genes are in a region of 3023 bp of identical genomic sequence, the two genes were therefore treated as one in all analyzes. ^h ^Classified as belonging to one of the Aquaporin KOG groups (KOG0223 or KOG0224) but without further description in PpDB ^i ^the complete comment is "Possibly an aquaporin, similar to NIP1;2, with one signature peptide, "HFNPAVSV"".

When this study was initiated only 11 out of the 23 PpMIPs had been described in the literature [20,40]. Since then one more of the 23 PpMIPs (PpPIP2;1) has been published [41]. All 23 PpMIP sequences were categorized as belonging to an aquaporin euKaryotic Orthologous Groups (KOG) at the PpDB and most of these also had a suggested classification (Table 1). Based on the phylogeny of the PpMIPs together with the AtMIPs and *Z. mays *MIPs (ZmMIPs) a new and more systematic classification of the PpMIPs, that is consistent with the AtMIPs and ZmMIPs nomenclature [16,18], is proposed (Table 1).

### Phylogeny and classification

**Figure 1:**
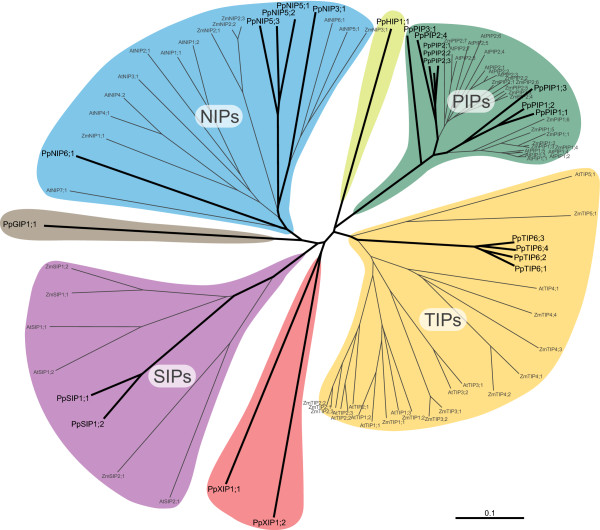
**Evolutionary relationship of plant MIPs**. An unrooted neighbour-joining tree showing the phylogenetic comparison of the complete set of 23 different MIPs from *P. patens *(Pp) in bold and the 35 respectively 33 MIPs from *A. thaliana *(At) and *Z. mays *(Zm). The seven subfamilies found in *P. patens *are indicated with the same colours as in Fig. 6. Note that the XIP, HIP and GIP have not been found in *A. thaliana *or *Z. mays*. The bar indicates the mean distance of 0.1 changes per amino acid residue.

**Figure 2:**
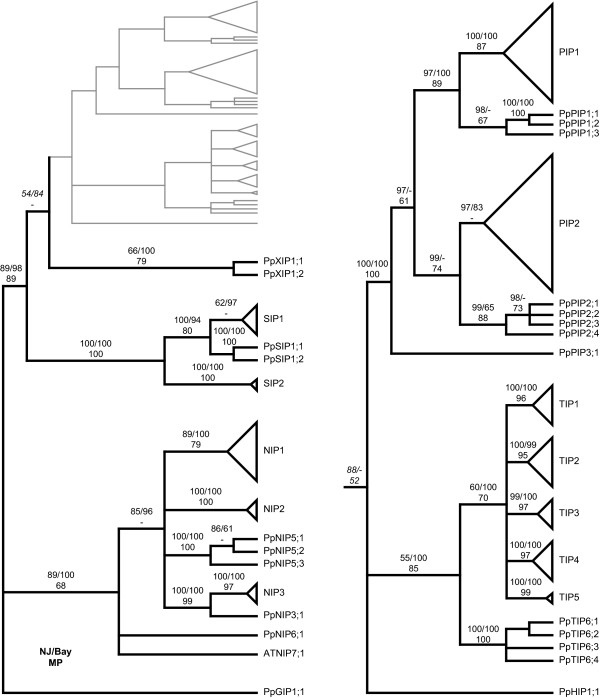
**Cladogram used for categorization of PpMIPs**. A "method consensus" cladogram, summarizing the overall robustness, as measured by bootstrapping for the neighbour joining (NJ) and maximum parsimony (MP) methods and posterior probabilities for the Bayesian (Bay) method. The tree was used for classification of the PpMIPs. The right panel shows an enlargement of the upper half of the tree. Note the low level of support (in italics) for the nodes basal to the PpHIP1;1 and the PpXIP-group, indicating the uncertainty of the placement of these groups. All nodes that have a support of less than 50 % for more than one method were collapsed. For visibility reasons, topology of clades with only *A. thaliana *and/or *Z. mays *MIPs are left out and replaced with triangles indicating the group. Support values for branches are presented as percentage, in the order NJ/Bay and underneath MP. A dash (-) indicates a support value of less than 50 %.

Using the full length protein alignments of all PpMIPs, AtMIPs and ZmMIPs [see Additional file 1] the neighbour joining (NJ) method resulted in one tree (Fig. 1) which was compared to trees from the maximum parsimony (MP) method and the Bayesian (Bay) method. Bootstrap support and Bayesian posterior probabilities were used to construct a "method-consensus" cladogram summarizing the results of the three methods and used to classify the PpMIPs (Fig. 2). The classification of AtMIPs and ZmMIPs in subgroups within subfamilies is similar for all MIPs except the NIPs. We named the PpNIPs according to the nomenclature used in classification of the NIPs in *Z. mays *and *O. sativa *since these four wider subgroups allow more sequence divergence and hence are more generic than the more narrow seven subgroups defined in *A. thaliana*. *P. patens *subgroups that failed to group with the previously classified subfamily groups were given consecutive higher indices (e.g. PpPIP3, PpTIP6, PpNIP5 or PpNIP6). In total 3 PpPIP1s, 4 PpPIP2s, 1 PpPIP3, 4 PpTIP6s, 1 PpNIP3, 3 PpNIP5s, 1 PpNIP6 and 2 PpSIP1s were categorized. Four PpMIPs failed to be classified into a subfamily, since they lack orthologs among the MIPs identified in *A. thaliana *and *Z. mays*. One of these was the MIP xenolog (homolog resulting from horizontal gene transfer) PpGIP1;1 previously identified as a GlpF-like MIP and named accordingly [20]. The remaining three were the PpHIP1;1 which shares similarities with both TIPs and PIPs but forms a separate distinct subfamily of its own, and the PpXIP1;1 and PpXIP1;2, two divergent MIPs that share some unique previously undescribed motifs.

To find orthologs of the three uncategorized PpMIPs (PpHIP1;1, PpXIP1;1 and PpXIP1;2) searches of databases at NCBI and embl were conducted. Hits representing a wide variety of species were selected and the corresponding protein sequences were aligned with the PpPIPs, the PpTIPs and either PpHIP1;1 or PpXIP1;1 and PpXIP1;2. The alignments were used in phylogenetic analyses to evaluate if the newly acquired sequences could help in categorizing the three PpMIPs. The PpHIP1;1 hits were mainly annotated as TIPs or AQP4s in the databases and the phylogenetic analysis resulted in three clusters (PIPs, TIPs and AQP4s) but PpHIP1;1 were still basal to all of these and could therefore not be assigned to any of these subfamilies (data not shown). As for PpXIP1;1 and PpXIP1;2, hits were mostly annotated as Plant MIP, TIP or AQP0 sequences. The phylogenetic analysis resulted in four different subfamilies, TIPs, PIPs AQP0s and a fourth clade consisting of unspecified plant MIPs and the PpXIPs (data not shown), see further analyses in next paragraph.

### The XIPs – an unrecognized MIP subfamily in higher plants

**Figure 3:**
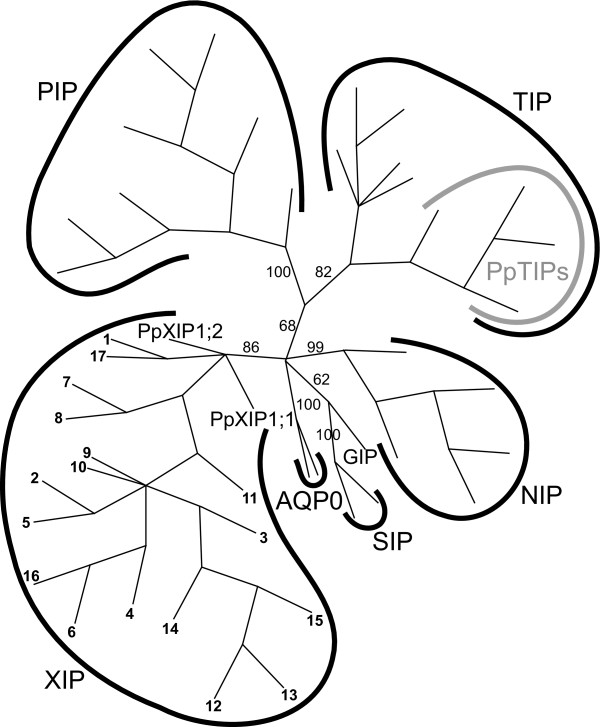
**Phylogenetic tree showing that the XIPs constitute a monophyletic subfamily distinct from other MIP subfamilies**. The unrooted bootstrap majority-rule consensus tree was generated with the parsimony method. Bootstrap support values in percentage are presented for the branches separating the subfamilies. The taxa in the XIP group are numbered for identification in Table 2. Except for these sequences and all PpMIPs (except PpHIP1;1), AQP0 sequences of *Bos taurus *[GenBank:NM_173937] and *Ovis aries *[GenBank:AY573927] and TIP sequences from *Picea abies *[GenBank:AJ005078], *Lotus japonicus *[GenBank:AF275315], *Helianthus annus *[GenBank:EF469912], *Oryza sativa *[GenBank:AB114829] and *Posidonia oceanica *[GenBank:AJ314583] were used.

Sequences belonging to this fourth clade have a weak overall sequence similarity to MIPs in general (about 30 % amino acid identity, data not shown), and could neither be assigned to any of the previously identified classes of plant MIPs (PIPs, TIPs, NIPs, SIPs and GIPs) nor be associated with the PpHIP1;1 sequence. However, some conserved motifs within this new subfamily (see discussion) were identified and based on these one representative sequence (the castor bean cDNA sequence [GenBank:EG656577]) was selected. This sequence was used in database searches in order to obtain more MIPs belonging to this novel subfamily. A handful of more sequences that all shared the same conserved motifs were identified. One of these sequences originated from *Populus trichocarpa *and therefore the *P. trichocarpa *genome at JGI were searched, identifying 4 more paralogs (Table 2). These sequences, together with the sequences retrieved from the castor bean cDNA and the PpXIP searches and all PpMIP sequences (except PpHIP1;1) were combined into one sequence alignment used in phylogenetic analysis. The resulting trees confirmed that the unclassified MIPs form a distinct monophyletic clade (with the PpXIPs as basal taxa), different from the other MIPs included in the analysis (Fig. 3). As shown in Table 3 there is considerable variation both at the first NPA box and the ar/R filter among the sequences in this clade. We propose that, awaiting further characterization, MIPs in the new subfamily should be referred to as X Intrinsic Proteins (XIPs) emphasizing that currently we have very little information on the function of these proteins.

**Table 2:** Sequences identified as belonging to the novel XIP subfamily

**Number**^a^	**ID**^b^	**Type**^c^	**Organism**	**Descr.**	**Comments**
1	DN837617	EST	*Selaginella moellendorffii*	-	cDNA from whole plant
2	BT014197	EST	*Solanum lycopersicum*^d^	-	cDNA from fruit
3	DY275505	EST	*Citrus clementina*	-	cDNA from mixed tissue
4	CO092422	EST	*Gossypium raimondii*	-	cDNA from whole seedlings
5	CK295158	EST	*Nicotiana benthamiana*	-	cDNA from mixed tissue
6	EG656577	EST	*Ricinus communis*	-	cDNA from seeds
7	EG666650	EST	*Ricinus communis*	-	cDNA from roots
8	CK746370^e ^DT60037^e^	EST	*Liriodendron tulipifera*	-	cDNA from flower buds
9	DR936893^e ^DT742029^e^	EST	*Aquilegia Formosa × Aquilegia pubescens*	-	cDNA from mixed tissue
10	AM455454	WGSS	*Vitis vinifera*	-	Exons between nucleotides 61100–61186, 61265–61354 & 61465–62185
11	AM455454	WGSS	*Vitis vinifera*	-	Exons between nucleotides 69471–69617 & 69685–70443
12	557139	Gene	*Populus trichocarpa*	PIP	no EST support
13	829126	Gene	*Populus trichocarpa*	PIP	EST support from cambium
14	767334	Gene	*Populus trichocarpa*	PIP	no EST support
15	759781	Gene	*Populus trichocarpa*	PIP	no EST support
16	821124	Gene	*Populus trichocarpa*	PIP	EST support from petioles
17	XM_639170	Gene	*Dictyostelium discoideum AX4 *^f^	MIP	Hypothetical protein

^a^Number used for identification in Fig. 3 ^b ^GenBank ID or Protein ID *for Populus trichocarpa v 1.1 *database at JGI ^c ^EST = Expressed Sequence Tag, WGSS = Whole Genome Shotgun Sequence, Gene = Annotated gene ^d ^Tomato, previously named *Lycopersicon esculentum *^e ^Two overlapping sequences were used to construct a full length sequence ^f ^The only non-plant species and a very divergent sequence

**Table 3:** Aromatic/arginine filter of PpMIPs and MIPs of the XIP subfamily

	**NPA motifs**		**Ar/R selectivity filter**^a^				
		
**MIP protein(s)**^b^	**Loop B**	**Loop E**	**H2**	**H5**	**LE**_1_	**LE**_2_	**Alt. H5**^c^
PpPIPs	NPA	NPA	F	H	T	R	
PpTIPs	NPA	NPG	H	I	A	R	
PpNIP3.1	NPA	NPV	A	I	A	R	
PpNIP5s	NPA	NPA	F	A	A	R	
PpNIP6.1	NPA	NPM	G	V	A	R	
PpSIPs	NPT	NPA	V	V	P	N	
PpGIP1.1	NPA	NPA	F	V	P	R	
PpHIP1.1	NPA	NPA	H	H	A	R	
PpXIP1.1	NPC	NPA	Q	A	A	R	A
PpXIP1.2	NPS	NPA	Q	I	A	R	Q
DN837617	NPI	NPA	L	Q	A	R	S
DY275505	NPL	NPA	V	V	A	R	T
AM455454.1	NPV	NPA	V	V	A	R	T
557139	NPI	NPA	V	V	A	R	T
829126	NPI	NPA	V	V	A	R	T
759781	NPI	NPA	V	V	A	R	T
EG666650	SPT	NPA	V	V	V	R	T
DR936893 DT742029	NPT	NPS	V	V	V	R	S
CK746370 D T60037	NPI	NPA	V	I	V	R	G
767334	NPL	NPA	A	V	A	R	T
CK295158	NPV	NPA	I	V	A	R	T
BT014197	NPV	NPA	I	V	A	R	T
AM455454.2	NPI	NPA	I	V	A	R	T
821124	NPA	NPA	I	V	V	R	T
EG656577	NPV	NPA	I	V	V	R	T
CO092422	NPV	NPA	I	V	V	R	T
XM_639170	NPS	NPA	H	S	F	R	I

^a ^The ar/R filter is defined by four amino acid residues: one in helix 2, one in helix 5 and two in loop E ^b ^The PpMIPs are identified with their proposed names and the other MIPs are identified by their GenBank accession numbers ^c ^Alternative residue at H5 position due to alignment of conserved glycines in helix 5, however this also introduces two extra amino acids between helix 5 and the second NPA box

### Gene structure

**Figure 4:**
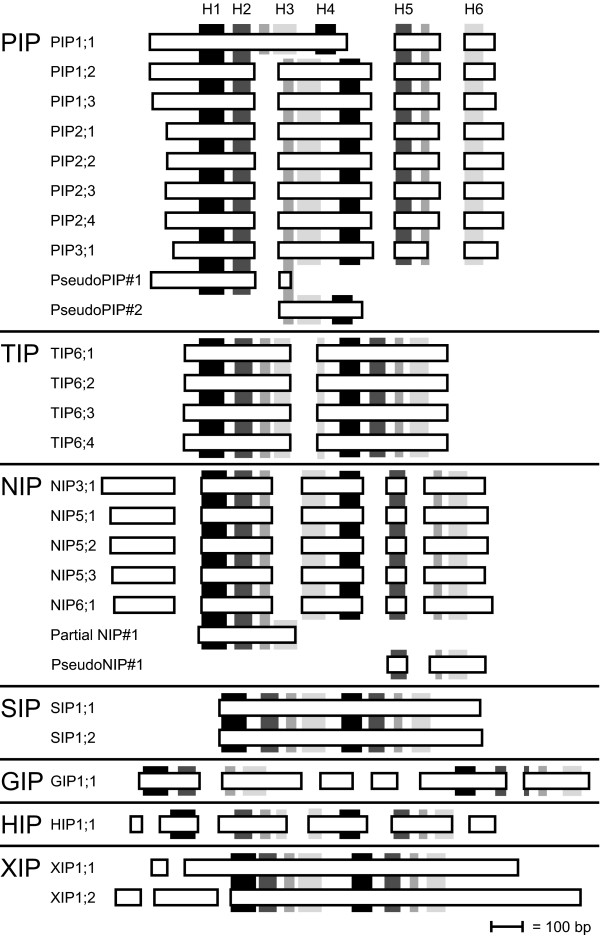
**The conserved structure of *MIP *genes in *P. patens *is consistent with their phylogenetic classification**. Horizontal bars represents exons (only coding sequence), gaps being introns. Position of transmembrane helices H1 to H6, and the two half transmembrane helices HB and HE, is indicated by vertical bars. Shading of the vertical bars shows the homologous helices in the first and second halves of the MIPs. Exons and transmembrane helices as well as position of transmembrane helices are drawn to scale, but introns are only depicted schematically, the bar indicates the length of 100 bp.

The average *PpMIP *was found to have 2.6 introns with a size of 246.4 bp. This is about half the number of introns, but of approximately the same size as predicted for the average *P. patens *gene in a genome wide analysis [42]. The exon/intron patterns of the *PpMIPs *were found to be highly conserved within each subfamily, as shown in Figure 4. Comparison with the *AtMIPs *showed the intron positions to be conserved for both *PIPs *and *NIPs*, but not for *TIPs *(in *P. patens *the intron position is 35 base pairs further to the 5'-end) and *SIPs *(completely lacking introns in *P. patens*). The exon/intron pattern also supported that the *PpHIP *and the *PpXIPs *were to be classified neither as *PIPs*, *TIPs*, *NIPs*, *SIPs *nor *GIPs*, but rather as separate subfamilies on their own.

The identification of five *P. trichocarpa *XIP paralogs allowed comparison of gene structure across species. All five *P. trichocarpa *genes have the same pattern of exon-introns with two introns in the N-terminal sequence (data not shown). This is also true for the PpXIP1;2, but since the N-termini have a high degree of interspecies variation it is hard to make any conclusion on whether the intron positions are exactly conserved.

## Discussion

### *Physcomitrella patens *Major Intrinsic Proteins

Comparison of protein superfamilies of distantly related species can aid in our understanding of protein function and by annotating all MIPs in *P. patens *we have made such a comparison possible for the MIP superfamily of higher plants and mosses. Originally we hypothesised that mosses were to have a relatively small superfamily, due to them being simpler (for example lacking vascular tissue and therefore having a less complex water transport regulation). It was therefore much to our surprise that we found *P. patens *to have seven subfamilies containing in total 23 different MIPs, an unexpected large and divergent superfamily. One of these (PpGIP1;1) is analysed in detail by Gustavsson et al. [20], and is therefore omitted from this discussion. Half of the remaining 22 PpMIPs are previously described by Borstlap [40] and Lienard et al. [41] and the remaining 11 are previously not described in the literature. The gene structure of the PpMIPs supports the phylogenetic analyses and the resulting division into seven subfamilies. Comparison with AtMIPs shows that PIPs and NIPs have conserved intron positions whereas SIPs and TIPs do not. This is consistent with the conservation of individual groups of the NIP and PIP subfamily in both *P. patens *and *A. thaliana *(discussed further below).

### PIPs – the most conserved MIPs in plants

PIPs are remarkably well conserved plant MIPs that can be further classified into PIP1s and PIP2s. Both PIP1s and PIP2s are highly conserved in *P. patens *indicating that these groups must have formed early on in the evolution of land plants and are of fundamental importance in plant physiology. The physiological relevance of PIP1s and PIP2s in water relations in higher plants is well established and recently also carbon dioxide has been added to the list of possible substrates [reviewed in [4]]. The ar/R filter is strictly conserved in PIPs including PpPIPs suggesting that all PIPs, irrespectively of subgroup, have the same substrate specificity (Table 3). It is likely that the evolution of PIP sequences is constrained also in many other ways. For example the PIPs reside in the plasma membrane and it is essential that they are impermeable for protons in order to maintain the proton gradient. Furthermore, the water permeability of PIPs can be regulated by phosphorylations, pH and Ca^2+ ^via an intricate gating mechanism [11]. From our results presented here it is clear that the diacidic motif in the N-terminal region and the histidine in the D-loop responsible for Ca^2+ ^binding and pH gating, respectively, are both conserved in all PpPIP1s and PpPIP2s. The phosphorylation site in loop B is also conserved in all PpPIPs whereas the PIP2 specific C-terminal phosphorylation motif is restricted to the PpPIP2s. This suggests that the gating mechanism is generic in all species and tissues where PIPs are expressed and that for instance pH gating is not limited to anaerobic conditions in roots of higher plants.

In *P. patens *there is also an odd PIP (PpPIP3;1), basal to both PIP1s and PIP2s. The PpPIP3;1 has a deletion of 11 amino acids after the second NPA-box (between helix E and helix 6) and this, together with the relatively high divergence from other PIPs (e.g. lack of the Ca^2+ ^binding site at the N terminal region and a conserved cysteine at helix 2) and the absence of ESTs, makes it questionable if this *MIP *gene is at all functional.

### TIPs specialization occurred later

It has already been suggested that *P. patens *is lacking the specific isoforms of TIPs observed in higher plants [40] and now, with this complete set of PpMIPs at hand, this is confirmed. Interestingly, it has been proposed that vacuole sub-types harbor specific sets of TIP isoforms [43] and it is easy to speculate that the TIP groups in higher plants evolved due to special functional requirements of different vacuoles. The identification of conserved proteins in *P. patens*, involved in the sorting of proteins to different types of vacuoles, suggests that there are most likely more than one type of vacuole in bryophytes [44]. This implies that TIPs are not conserved markers for subtypes of vacuoles as the presence of only one group of TIPs in *P. patens *indicates that either there is only one of the vacuole types in moss that has TIPs, or alternatively several different vacuoles in the moss cell all have the same type of TIPs. Both interpretations are consistent with recent experiments in higher plants that have challenged the idea of TIPs as valid markers for vacuole sub-types [45,46].

Rather than forming a very distant subclass of TIPs, the PpTIP6s appears as a conserved mosaic of the different motifs that are found in the different TIP groups of higher plants. For example the first few amino acid residues at the N-terminus are similar to TIP2s, whereas the C-terminal region is most similar to TIP3s. The identities of the amino acid residues at the ar/R filter (HIAR) are shared with both some TIP3s and TIP4s suggesting a similar specificity. In fact exactly these residues are the most common, comparing the frequencies in the selectivity regions of all *A. thaliana*, *Z. mays *and *O. sativa *TIPs (H_0.81_I_0.62_A_0.72_R_0.75_; based on Table 4 in [47]). This makes it likely that PpTIP6s are similar to the TIPs present in the last common ancestor of bryophytes and vascular plants and that the other motifs found at these positions are derived characters that have appeared later as different groups of TIPs evolved in vascular plants. The expansion and formation of specialized groups in the TIP subfamily of higher plants might suggest that some of these TIPs have taken over the functions of the MIPs of subfamilies that are missing in higher plants (e.g. HIPs and XIPs).

### NIP groups evolved early

In higher plants NIPs form a divergent subfamily with large variation between species. This is true also for NIPs in *P. patens*, but surprisingly one of the three NIP groups identified is present also in higher plants, indicating that this group of NIPs, NIP3, was present already in a common ancestor to *P. patens *and higher plants (Fig. 2). The conserved intron positions among *NIPs *in *A. thaliana *and *P. patens *indicate that this gene structure was also present in the ancestral *NIP *gene. NIPs are different from other MIPs in that they often have unorthodox NPA boxes. In many NIP3s of higher plants the first and second NPA boxes are replaced by NPS and NPV, respectively [47]. The corresponding motifs in PpNIP3;1 are NPA and NPV (Table 3), which is identical to AtNIP6;1 (one of the two NIP3s in *A. thaliana *according to the monocot classification), suggesting that NIP3s had these motifs before the split of bryophytes and vascular plants.

The two NIP groups specific for *P. patens *(PpNIP5 and PpNIP6), have a unique combination of amino acids at the ar/R filter (Table 3). In contrast the ar/R region of PpNIP3;1 conforms to the residues found in other NIP3s, supporting that they are orthologs with the same conserved function. Recently a NIP3 have been shown to have a role in boron uptake in roots of *A. thaliana *[27] and even though mosses lack roots it cannot be ruled out that PpNIP3;1 has a role in boron transport in the moss.

The N-terminal region of NIPs is relatively long compared to most other plant MIPs and is encoded on a separate exon. Due to the lack of generally conserved motifs in this region the first exon is often missing in annotations of *NIP *genes. However, within NIP3s of higher plants several motifs have been recognized in the N-terminal region [48] and some of these features are also conserved in PpNIP3;1. Similar to higher plants PpNIP3;1 has a high degree of proline and threonine residues and a sequence (AKCFP), corresponding to the conserved motif (C [KN]C [LF] [PS]) in higher plants.

Many NIPs in higher plants have a conserved potential phosphorylation motif in the C-terminal region corresponding to the phosphorylation site in *Glycine max *NOD26 (GmNOD26, S262) and *Spinacia oleracea *PIP2;1 (SoPIP2;1; S274) [5,49]. A serine at this position is also present in a similar motif in NIP3s of higher plants ([RK]XXR**S**FXR) [48] but not in PpNIP3;1 where the serine is substituted to a valine. In PpNIP5;3 and PpNIP6;1 there are serines but some of the basic residues in the motif are not conserved. In contrast a corresponding serine in the motif (KXXK**S**F [HR]R) is present in PpNIP5;1 and PpNIP5;2 suggesting that at least some NIPs in a common ancestor of bryophytes and higher plants were regulated by phosphorylation.

It is interesting to see that there is no NIP2 type of MIP in *P. patens*, a NIP-group recently identified as a silicon transporter in rice [28]. Since bryophytes are known to accumulate silicon [50], the lack of PpNIP2s suggests that this function is carried out by a different isoform or class of proteins in *P. patens*.

### Only SIP1s are found in *Physcomitrella patens*

In *A. thaliana *there are two classes of SIPs, SIP1s and SIP2s, both having the same gene structure with two introns at conserved positions [16]. In *P. patens *there are two SIPs but neither of them has an intron. Surprisingly both of the PpSIPs belong to the SIP1 group whereas SIP2s of higher plants form a basal clade. This suggests that either SIP2s were present already in early land plants but were subsequently lost in *P. patens *in which the remaining SIP1s were subject to intron loss, or that SIP2s have rapidly diverged from SIP1s after the split leading to mosses and higher plants. An intron loss in *PpSIP1s *or an intron gain in a common ancestor to *SIP1s *and *SIP2s *in higher plant is equally likely in this scenario. In most SIP1s the corresponding sequence to the first NPA box is NPT, interestingly this unusual motif is conserved also in PpSIP1s, implying that this is a structurally and functionally important feature of SIP1s. In addition the ar/R filter is consistent with the phylogenetic classification, suggesting a conserved function of SIP1s among terrestrial plants.

### HIP a unique MIP with similarities to both PIPs and TIPs

There are three *P. patens *MIP sequences that cannot be classified into any of the five subfamilies previously described in plants [16,20]. One of these, the PpHIP1;1, seems to be a rather rare MIP, since we were not able to identify any orthologs. The unique gene structure indicates that this protein belongs to a separate subfamily. In phylogenetic analyses PpHIP1;1 tend to cluster with PIPs and TIPs, although the support for this is not very strong as seen in Figure 2. Upon looking at the ar/R filter (Table 3) one could also speculate that the HIP is related to TIPs and PIPs, since it has histidines both at the H2 position, typical for TIPs and the H5 position, typical for PIPs. What effect having two large and basic amino acid residues in the filter will have on transport properties is however unclear, and since there are no ESTs of the gene it might even be that it is not expressed. According to a subcellular localization prediction (WoLF PSORT [51], data not shown) PpHIP1;1 is slightly more likely to reside in the tonoplast than the plasma membrane. Further studies are required to explore expression, localization and substrate specificity of the PpHIP.

The two other sequences belong to another group, the XIPs, further discussed in the next paragraph.

### The XIP subfamily

A search for PpXIP orthologs resulted in the finding of many XIP sequences from a wide variety of species, including five paralogs from *P. trichocarpa (*probably the same five described as "putative aquaporins lacking in the *Arabidopsis*" by Tuskan et al. [52]). It is striking that no sequences are from monocots. Although most sequences were from dicots, no ortholog was found in *A. thaliana*, which may be explained by gene loss due to a relatively recent reduction of the genome size [53]. Phylogenetic analyses confirmed that these sequences are from a, to our knowledge, previously unrecognized MIP subfamily, different from PIPs, TIPs, NIPs, SIPs and GIPs. The only non-plant sequence included in the analyses was a protein encoded by the [GenBank:XM_639170] gene from the amoeba *Dictyostelium discoideum AX4 *and it should be pointed out that although this protein is clustering with the XIPs in phylogenetic analyses, it is annotated as a hypothetical protein and lacks some of the characteristics of the XIPs. For example the amoeba protein has NPA boxes and an ar/R filter different from all other XIPs and also an overall highly divergent MIP sequence, all which makes it questionable if this protein has the same function as other XIPs. There is also a sequence from a lycophyte, the spike moss *Selaginella moellendorffii*, which together with the two PpXIPs are the three most divergent sequences albeit all three are clearly categorisable as XIPs. Although most sequences were derived from ESTs, no general conclusion could be made on expression pattern, since XIP transcripts were isolated from many different tissues ranging from roots, seedlings, flower buds to seeds and fruits (Table 2). Based on a subcellular localization prediction XIPs are likely to be situated in the plasma membrane (WoLF PSORT [51], data not shown).

**Figure 5:**
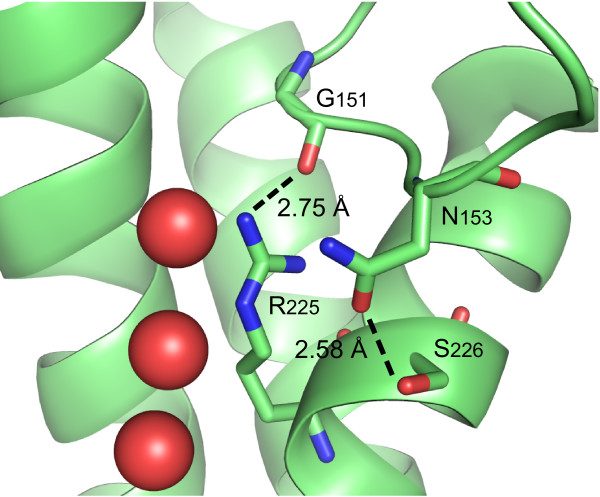
**Interaction of loop C and helix E**. Detail from the structure of SoPIP2;1 illustrating how loop C and residues in helix E interacts via H-bonds. In XIPs N153 and S226 are replaced by cysteins suggesting a covalent linkage between loop C and helix E. Oxygens of water molecules at the ar/R region are represented by spheres and the discussed residues are depicted by sticks.

In the first NPA box of the XIPs, the alanine is replaced by a valine, leucine, isoleucine, serine or cysteine. All of these replacements, except isoleucine, have been observed in NPA boxes of other MIPs [47]. The most conserved feature of the new subfamily is located after the second NPA box, where a cysteine amino acid is thoroughly conserved in the motif NPAR**C**. This cysteine is only a moderate change of the conserved serine or threonine found in many other subfamilies e.g. PIPs, TIPs, NIPs and in several mammalian AQPs. However, from the solved structure of SoPIP2;1 it is clear that residues at this position can stabilize the conformation of the C-loop by hydrogen bonds ([PDB:1Z98];S226 – N153, see Fig. 5) an interaction that seem to be structurally conserved and that also can be seen in BtAQP1 ([PDB:1J4N]; S198 – N129), BtAQP0 ([PDB:1YMG];S188 - N119) and, with the donor-acceptor interchanged, in EcGlpF ([PDB:1FX8];D207 - T137). This stabilisation is probably directly affecting the permeability of the pore since the orientation of the arginine of the ar/R filter is also stabilised by a hydrogen bond to the backbone of the C-loop (Fig. 5). Interestingly all the XIPs also have a conserved cysteine resulting in the motif LGG**C **in the C-loop at a position that can be aligned to N153 in SoPIP2;1. This suggests that a cysteine bridge may covalently fixate the C-loop relative to the arginine in the XIPs and that the extracellular entrance to the pore therefore might be more rigid than that of other MIPs.

There is also a highly conserved motif with a proline at the end of helix 2, 7 amino acids before the first NPA-box (**P**ISGGHINP), also found in mammalian AQP5s. A corresponding motif can be found in helix 5 of many other plant MIPs, which is interesting as this reflects the symmetry of the MIP proteins, consisting of two direct repeats of sequence. It is also worth noting that, with the exception of PpXIPs, there is a lack of an otherwise highly conserved glycine in helix 5, allowing the close packing of helix 2 and 5 [54], which in most XIPs is replaced by either a leucine or an isoleucine. An alternative alignment that retains the conserved glycine, but introduces two extra amino acids between helix 5 and the second NPA box is possible, but not used in the analysis presented here. This alignment will also affect which amino acid is positioned in the H5 position of the ar/R filter (Table 3). In the chosen alignment a valine is the most frequent residue in the H5 position and in the alternative alignment threonine would be in the H5 position. At the H2 position most XIPs have an aliphatic amino acid, something that can also be found in some NIPs and SIPs [47]. This suggests that XIPs are not primarily water channels, although substrate specificity experiments have to be carried out to establish this. In the XIPs from *P. patens *and *S. moellendorffii *there is a glutamine at the H2 respectively H5 position of the ar/R filter, also found in TIP4s and TIP5s of higher plants, suggesting that maybe these TIPs have taken over some function of the XIPs in primitive plants. Further studies of localization, specificity and expression patterns are needed in order to determine the function of this novel MIP subfamily.

## Conclusion

**Figure 6:**
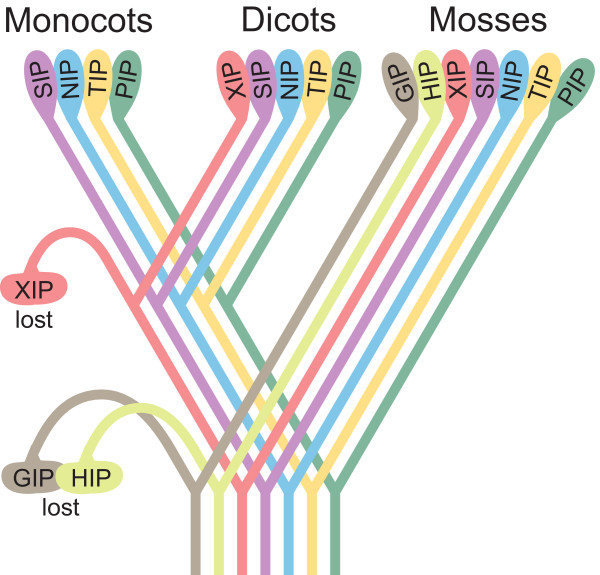
**The evolution of the MIP superfamily in plants**. A schematic drawing of a likely scenario for the evolution of the MIP superfamily in plants. The ancestral plant is proposed to have had all seven subfamilies of MIPs found in extant mosses. The GIP and HIP were lost during the evolution of higher plants and subsequently the XIP subfamily was lost in monocots.

In this study we identified a surprisingly large number of MIP encoding genes in *P. patens*, forming a diverse superfamily with seven subfamilies. In total 23 PpMIPs were identified; eight PIPs, four TIPs, five NIPs and two SIPs, one GIP and three MIPs belonging to two different, novel subfamilies, the HIPs and the XIPs. HIPs are hitherto not found in any higher plants, whereas the XIPs seem to be present in many plant species, although not in monocots. Interestingly, specific groups within the subfamilies, like PIP1s, PIP2s, NIP3s and possibly SIP1s were already present in a common ancestor of higher plants and bryophytes. In contrast, the subgroups of TIPs probably evolved later. These results suggest that early land plants had a large and divergent MIP superfamily consisting of at least the seven subfamilies found in *P. patens *and that during the evolution of higher plants some subfamilies were lost (Fig. 6) whereas remaining subfamilies evolved further resulting in diversification and formation of subgroups within the subfamilies. We speculate that some of the new subgroups, or perhaps some other unrelated transporters have taken over the function of the lost MIP subfamilies in higher plants.

## Methods

### Gene identification and annotation

*Physcomitrella patens MIP *genes were identified by TBLASTN searches of the PpDB at the Joint Genome Institute [37] using the protein sequences of the complete set of 35 MIPs from *Arabidopsis thaliana *as queries [16]. Gene models overlapping with hits were manually inspected and kept based on subfamily sequence similarity or EST support. If no satisfying model existed, the genomic sequence was used to identify exons for the new or modified model (as specified in Table 1). The PpGIP1;1 sequence was also added to the sequences since it was previously identified as a PpMIP [20]. Protein sequences corresponding to the translation of the *PpMIP *genes were used in a second round of TBLASTN searches to identify more divergent MIP sequences in PpDB, but none were found. The resulting 23 PpMIPs were used in a multiple alignment of translated sequences, together with the 35 AtMIP and 33 ZmMIPs [18]. Alignments were manually inspected and adjusted and care was taken to keep the number of gaps low and to avoid gaps in functionally important features, such as the NPA-boxes and transmembrane regions. The alignment that forms the basis for all the phylogenetic analysis regarding the PpMIPs presented here is available as ALIGN_001168 in the EMBL-align database (which can be accessed either via the EMBL-EBI SRS homepage [55] or FTP [56]).

Orthologs of the unclassified PpHIP, PpXIP1;1 and PpXIP1;2 were searched for by TFASTX3 searches of the EMBL nucleotide sequence database [57] and TBLASTN searches of the nr/nt, est, gss and htgs databases at NCBI [58] using the translated sequence of the three *PpMIP*s. Translations representing hits from a wide variety of species were used in protein alignments together with either PpHIP1;1 or PpXIP1;1 and PpXIP1;2 and the PpPIPs and PpTIPs. The alignments were manually inspected and adjusted as mentioned above and used for phylogenetic analysis of PpHIP1;1 and the PpXIPs and are available in the EMBL-align database as ALIGN_001169 respectively ALIGN_001170.

The translated sequence of one of the PpXIP orthologs found [GenBank:EG656577] was used in additional TBLASTN searches of the nr/nt, est, gss and htgs databases at NCBI in order to find more homologs of this group. One ortholog found was from *Populus trichocarpa *and a translation of this sequence was used in a TBLASTN search of the *P. trichocarpa *genome at JGI to find paralogs. These paralogs together with a selection of homologs from the [GenBank:EG656577] and PpXIP searches were used in a multiple sequence alignment of translated sequences together with 22 PpMIPs (all except the PpHIP). The alignment was manually inspected and adjusted in the same manner as the PpMIP-AtMIP-ZmMIP alignment. This alignment forms the basis for all the phylogenetic analysis regarding the XIP group of MIPs and is available as ALIGN_001171 in the EMBL-align database.

### Phylogenetic analysis

The PpMIP sequence alignment was analyzed by three different phylogenetic methods, Neighbour Joining (NJ), Maximum Parsimony (MP) and Bayesian inference (Bay). For all methods, gaps were treated as missing data. PAUP*4.0b10 [59] was used for the NJ and MP analysis. The default settings were used for both methods and bootstrapping with one thousand replicates for each method assessed the confidence of the best trees. Bayesian phylogenetic inferences were conducted using MrBayes 3.0.2 [60] using vague or uninformative prior probability distributions of the likelihood model under the JTT [61] +I+Γ model. Two sets of four parallel Metropolis Coupled Monte Carlo Markov Chains, of which three were heated with 0.2 temperature increments, were run for 2 million generations starting from random trees. Each 100th tree was sampled. The first 25 % of sampled trees was discarded as burn in, and stationary phase was empirically determined by looking at the likelihood scores of the kept samples. Robustness of the inferred tree was evaluated using Bayesian posterior probabilities. A "method consensus" tree was constructed as an overview, in this tree only branches that had a bootstrap or posterior probability support of more than 50 % in at least two of the methods were kept and all other were collapsed.

For the PpHIP1;1, PpXIPs and XIP-group alignments, PAUP*4.0b10 [59] was used for a NJ and MP analysis (gaps treated as missing data). The default settings were used for both methods and for the XIP-group alignment analysis, bootstrapping with one thousand replicates for each method assessed the confidence of the best trees. All trees from the PpMIP, PpHIP, PpXIPs and XIP family analyses are available in nexus format for viewing in Tree-View [62] [see Additional files 2, 3, 4, 5, 6, 7, 8, 9, 10, 11, 12, 13, 14].

## Authors' contributions

JÅHD carried out the acquisition, analysis and interpretation of data and drafting of the manuscript. UJ conceived the study and helped with the interpretation of data. Both authors worked with the design of the study and with revising the manuscript and they both read and approved the final manuscript.

## Note added in proof

During the publication of this work we successfully identified the HIP subfamily of MIPs in the spike moss *Selaginella moellendorffii*. PpHIP1;1 and the closest homolog in *S. moellendorffii *are highly similar (with 73.7 % amino acid identity) and have the same NPA-boxes and ar/R filter motives. This proves that the HIP subfamily is indeed a novel conserved subfamily of MIPs and not an anomaly only found in *Physcomitrella patens*.

## Supplementary Material

Additional file 1 Figure showing the alignment of PpMIPs, AtMIPs and ZmMIPs. Shading is indicating the degree of conservation of an amino acid at a position. The actual alignment is available as "ALIGN_001168" from the EMBL align database. Click here for file

Additional file 2 Phylogenetic tree (in nexus format) using the Bayesian inference method and the dataset ALIGN_001168. Click here for file

Additional file 3 Bootstrap majority consensus phylogenetic tree (in nexus format) using the Parsimony method and the dataset ALIGN_001168. Click here for file

Additional file 4 Phylogenetic tree (in nexus format) using the Parsimony method and the dataset ALIGN_001168. Click here for file

Additional file 5 Bootstrap majority consensus phylogenetic tree (in nexus format) using the Neighbour Joining method and the dataset ALIGN_001168. Click here for file

Additional file 6 Phylogenetic tree (in nexus format) using the Neighbour Joining method and the dataset ALIGN_001168. Click here for file

Additional file 7 Phylogenetic tree (in nexus format) using the Parsimony method and the dataset ALIGN_001169. Click here for file

Additional file 8 Phylogenetic tree (in nexus format) using the Neighbour Joining method and the dataset ALIGN_001169. Click here for file

Additional file 9 Phylogenetic tree (in nexus format) using the Parsimony method and the dataset ALIGN_001170. Click here for file

Additional file 10 Phylogenetic tree (in nexus format) using the Neighbour Joining method and the dataset ALIGN_001170. Click here for file

Additional file 11 Bootstrap majority consensus phylogenetic tree (in nexus format) using the Parsimony method and the dataset ALIGN_001171. Click here for file

Additional file 12 Phylogenetic tree (in nexus format) using the Parsimony method and the dataset ALIGN_001171. Click here for file

Additional file 13 Bootstrap majority consensus phylogenetic tree (in nexus format) using the Neighbour Joining method and the dataset ALIGN_001171. Click here for file

Additional file 14 Phylogenetic tree (in nexus format) using the Neighbour Joining method and the dataset ALIGN_001171. Click here for file
