# Supplementary material for: Unexpected complexity of the Aquaporin gene family in the moss Physcomitrella patens
Source: BMC Plant Biol. 2008 Apr 22;8:45. doi: 10.1186/1471-2229-8-45 (PMC2386804; doi:10.1186/1471-2229-8-45)
Supplement: Additional file 1 — Figure showing the alignment of PpMIPs, AtMIPs and ZmMIPs. Shading is indicating the degree of conservation of an amino acid at a position. The actual alignment is available as "ALIGN_001168" from the EMBL align database. [file 1471-2229-8-45-S1.pdf]

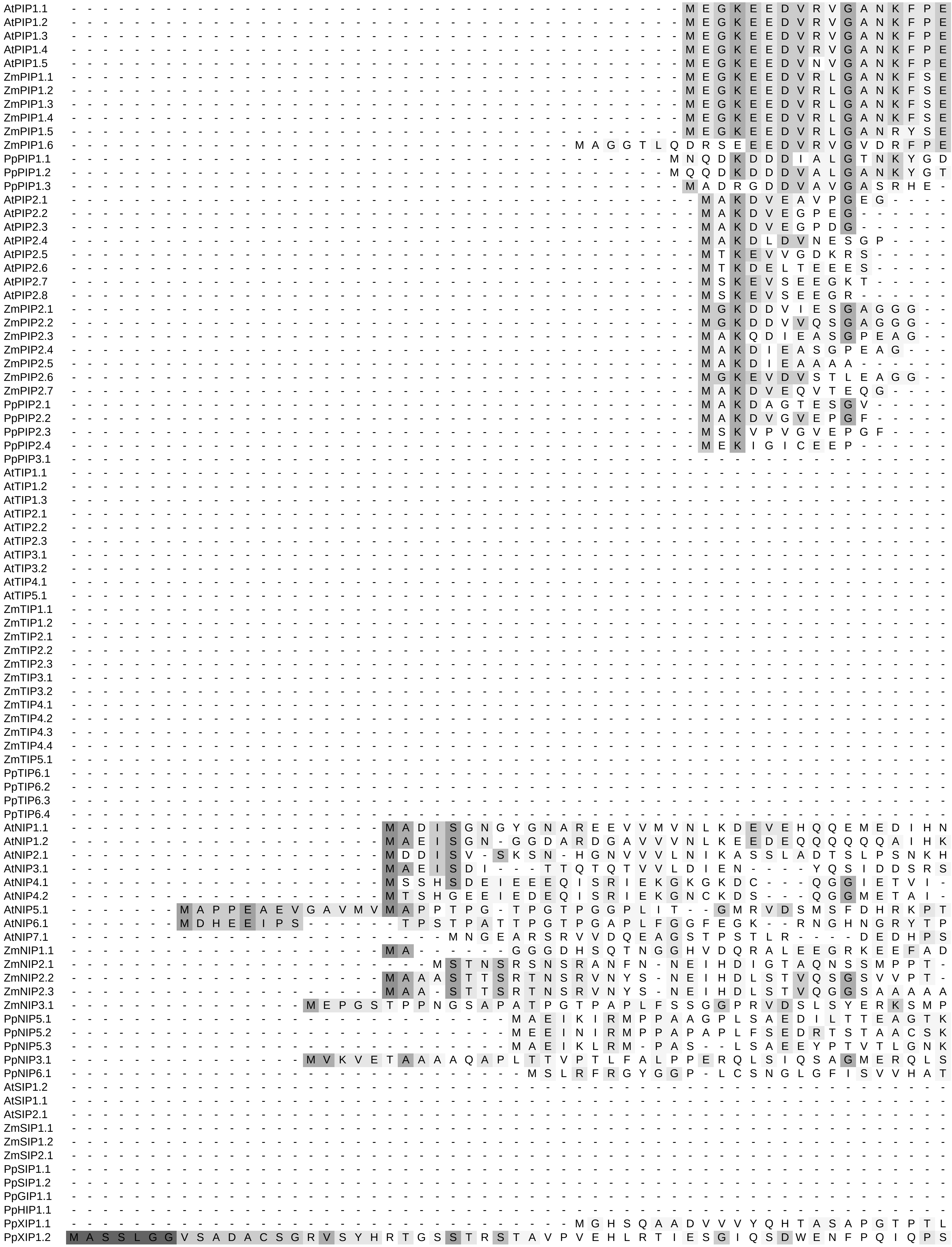

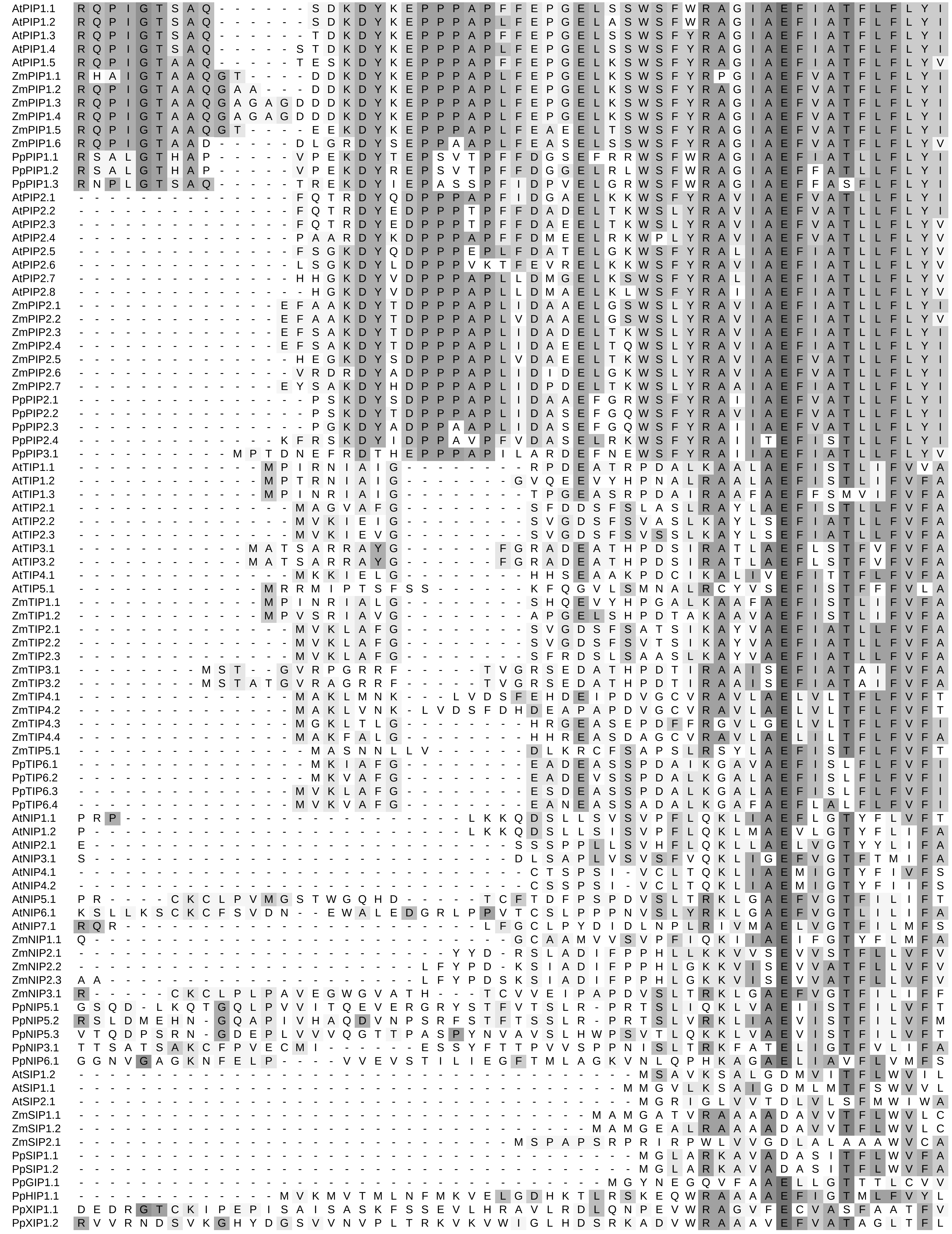
AtPIP1.1 RQPIGTSAQ------SDKDYKEPPPAPFFEPGELSSWSFWRAGIAEFIATFLFLYI
AtPIP1.2 RQPIGTSAQ------SDKDYKEPPPAPLFEPGELASWSFWRAGIAEFIATFLFLYI
AtPIP1.3 RQPIGTSAQ------TDKDYKEPPPAPFFEPGELSSWSFYRAGIAEFIATFLFLYI
AtPIP1.4 RQPIGTSAQ-----STDKDYKEPPPAPLFEPGELSSWSFYRAGIAEFIATFLFLYI
AtPIP1.5 RQPIGTAAQ-----TESKDYKEPPPAPLFEPGELKSWSFYRAGIAEFIATFLFLYV
ZmPIP1.1 RHAIGTAAQGT----DDKDYKEPPPAPLFEPGELKSWSFYRPGIAEFVATFLFLYI
ZmPIP1.2 RQPIGTAAQGAA---DDKDYKEPPPAPLFEPGELKSWSFYRAGIAEFVATFLFLYI
ZmPIP1.3 RQPIGTAAQGAGAGDDDKDYKEPPPAPLFEPGELKSWSFYRAGIAEFVATFLFLYI
ZmPIP1.4 RQPIGTAAQGAGAGDDDKDYKEPPPAPLFEPGELKSWSFYRAGIAEFVATFLFLYI
ZmPIP1.5 RQPIGTAAQGT----EEKDYKEPPPAPLFEAEELTSWSFYRAGIAEFVATFLFLYI
ZmPIP1.6 RQPIGTAAD------DLGRDYSEPPAAPLFEASELSSWSFYRAGIAEFVATFLFLYV
PpPIP1.1 RSALGTHAP-----VPEKDYTEPSVTPFFDGSEFRRWSFWRAGIAEFIATLLFLYI
PpPIP1.2 RSALGTHAP-----VPEKDYREPSVTPFFDGGELRLWSFWRAGIAEFFATLLFLYI
PpPIP1.3 RNPLGTSAQ-----TREKDYIEPASSPFIDPVELGRWSFWRAGIAEFFASFLFLYI
AtPIP2.1 --------------FQTRDYQDPPPAPFIDGAELKKWSFYRAVIAEFVATLLFLYI
AtPIP2.2 --------------FQTRDYEDPPPTPFFDADELTKWSLYRAVIAEFVATLLFLYI
AtPIP2.3 --------------FQTRDYEDPPPTPFFDAEELTKWSLYRAVIAEFVATLLFLYV
AtPIP2.4 --------------PAARDYKDPPPAPFFDMEELRKWPLYRAVIAEFVATLLFLYV
AtPIP2.5 --------------FSGKDYQDPPPEPLFDATELGKWSFYRALIAEFIATLLFLYV
AtPIP2.6 --------------LSGKDYLDPPPVKTFEVRELKKWSFYRAVIAEFIATLLFLYV
AtPIP2.7 --------------HHGKDYVDPPPAPLLDMGELKSWSFYRALIAEFIATLLFLYV
AtPIP2.8 ---------------HGKDYVDPPPAPLLDMAELKLWSFYRAIIAEFIATLLFLYV
ZmPIP2.1 -------------EFAAKDYTDPPPAPLIDAAELGSWSLYRAVIAEFIATLLFLYI
ZmPIP2.2 -------------EFAAKDYTDPPPAPLVDAAELGSWSLYRAVIAEFIATLLFLYV
ZmPIP2.3 -------------EFSAKDYTDPPPAPLIDADELTKWSLYRAVIAEFIATLLFLYI
ZmPIP2.4 -------------EFSAKDYTDPPPAPLIDAEELTQWSLYRAVIAEFIATLLFLYI
ZmPIP2.5 --------------HEGKDYSDPPPAPLVDAEELTKWSLYRAVIAEFVATLLFLYI
ZmPIP2.6 --------------VRDRDYADPPPAPLIDIDELGKWSLYRAVIAEFVATLLFLYI
ZmPIP2.7 -------------EYSAKDYHDPPPAPLIDPDELTKWSLYRAAIAEFIATLLFLYI
PpPIP2.1 ---------------PSKDYSDPPPAPLIDAAEFGRWSFYRAIIAEFVATLLFLYI
PpPIP2.2 ---------------PSKDYTDPPPAPLIDASEFGQWSFYRAVIAEFVATLLFLYI
PpPIP2.3 ---------------PGKDYADPPAAPLIDASEFGQWSFYRAIIAEFVATLLFLYI
PpPIP2.4 -------------KFRSKDYIDPPAVPFVDASELRKWSFYRAIITEFISTLLFLYI
PpPIP3.1 ----------MPTDNEFRDTHEPPPAPILARDEFNEWSFYRAIIAEFIATLLFLYV
AtTIP1.1 ------------MPIRNIAIG--------RPDEATRPDALKAALAEFISTLIFVVA
AtTIP1.2 ------------MPTRNIAIG-------GVQEEVYHPNALRAALAEFISTLIFVFA
AtTIP1.3 ------------MPINRIAIG--------TPGEASRPDAIRAAFAEFFSMVIFVFA
AtTIP2.1 --------------MAGVAFG--------SFDDSFSLASLRAYLAEFISTLLFVFA
AtTIP2.2 --------------MVKIEIG--------SVGDSFSVASLKAYLSEFIATLLFVFA
AtTIP2.3 --------------MVKIEVG--------SVGDSFSVSSLKAYLSEFIATLLFVFA
AtTIP3.1 -----------MATSARRAYG------FGRADEATHPDSIRATLAEFLSTFVFVFA
AtTIP3.2 -----------MATSARRAYG------FGRADEATHPDSIRATLAEFLSTFVFVFA
AtTIP4.1 --------------MKKIELG--------HHSEAAKPDCIKALIVEFITTFLFVFA
AtTIP5.1 ------------MRRMIPTSFSS------KFQGVLSMNALRCYVSEFISTFFFVLA
ZmTIP1.1 ------------MPINRIALG--------SHQEVYHPGALKAAFAEFISTLIFVFA
ZmTIP1.2 ------------MPVSRIAVG--------APGELSHPDTAKAAVAEFISTLIFVFA
ZmTIP2.1 --------------MVKLAFG--------SVGDSFSATSIKAYVAEFIATLLFVFA
ZmTIP2.2 --------------MVKLAFG--------SVGDSFSVTSIKAYVAEFIATLLFVFA
ZmTIP2.3 --------------MVKLAFG--------SFRDSLSAASLKAYVAEFIATLLFVFA
ZmTIP3.1 --------MST--GVRPGRRF-----TVGRSEDATHPDTIRAAISEFIATAIFVFA
ZmTIP3.2 --------MSTATGVRAGRRF-----TVGRSEDATHPDTIRAAISEFIATAIFVFA
ZmTIP4.1 --------------MAKLMNK---LVDSFEHDEIPDVGCVRAVLAELVLTFLFVFT
ZmTIP4.2 --------------MAKLVNK-LVDSFDHDEAPAPDVGCVRAVLAELVLTFLFVFT
ZmTIP4.3 --------------MGKLTLG--------HRGEASEPDFFRGVLGELVLTFLFVFI
ZmTIP4.4 --------------MAKFALG--------HHREASDAGCVRAVLAELILTFLFVFA
ZmTIP5.1 ---------------MASNNLLV------DLKRCFSAPSLRSYLAEFISTFLFVFT
PpTIP6.1 ---------------MKIAFG--------EADEASSPDAIKGAVAEFISLFLFVFI
PpTIP6.2 ---------------MKVAFG--------EADEVSSPDALKGALAEFISLFLFVFI
PpTIP6.3 --------------MVKLAFG--------ESDEASSPDALKGALAEFISLFLFVFI
PpTIP6.4 --------------MVKVAFG--------EANEASSADALKGAFAEFLALFLFVFI
AtNIP1.1 PRP----------------------LKKQDSLLSVSVPFLQKLIAEFLGTYFLVFT
AtNIP1.2 P------------------------LKKQDSLLSISVPFLQKLMAEVLGTYFLIFA
AtNIP2.1 E---------------------------SSSPPLLSVHFLQKLLAELVGTYYLIFA
AtNIP3.1 S---------------------------DLSAPLVSVSFVQKLIGEFVGTFTMIFA
AtNIP4.1 -----------------------------CTSPSI-VCLTQKLIAEMIGTYFIVFS
AtNIP4.2 -----------------------------CSSPSI-VCLTQKLIAEMIGTYFIIFS
AtNIP5.1 PR----CKCLPVMGSTWGQHD-----TCFTDFPSPDVSLTRKLGAEFVGTFILIFT
AtNIP6.1 KSLLKSCKCFSVDN--EWALEDGRLPPVTCSLPPPNVSLYRKLGAEFVGTLILIFA
AtNIP7.1 RQR-----------------------LFGCLPYDIDLNPLRIVMAELVGTFILMFS
ZmNIP1.1 Q---------------------------GCAAMVVSVPFIQKIIAEIFGTYFLMFA
ZmNIP2.1 ------------------------YYD-RSLADIFPPHLLKKVVSEVVSTFLLVFV
ZmNIP2.2 ----------------------LFYPD-KSIADIFPPHLGKKVISEVVATFLLVFV
ZmNIP2.3 AA--------------------LFYPDSKSIADIFPPHLGKKVISEVVATFLLVFV
ZmNIP3.1 R-----CKCLPLPAVEGWGVATH---TCVVEIPAPDVSLTRKLGAEFVGTFILIFF
PpNIP5.1 GSQD-LKQTGQLPVVITQEVERGRYSTFVTSLR-PRTSLIQKLVAEIISTFILVFT
PpNIP5.2 RSLDMEHN-GQAPIVHAQDVNPSRFSTFTSSLR-PRTSLVRKLIAEVISTFILVFM
PpNIP5.3 VTQDPSRN-GDEPLVVVQGTTPASPYNVAVSLHWPSVTLQKKLVAEVISTFILVFT
PpNIP3.1 TTSATSAKCFPVECMI------ESSYFTTPVVSPPNISLTRKFATELIGTFVLIFA
PpNIP6.1 GGNVGAGKNFELP----VVEVSTILIEGFTMLAGKVNLQPHKAGAELIAVFLVMFS
AtSIP1.2 ------------------------------------MSAVKSALGDMVITFLWVIL
AtSIP1.1 -----------------------------------MMGVLKSAIGDMLMTFSWVVL
AtSIP2.1 ------------------------------------MGRIGLVVTDLVLSFMWIWA
ZmSIP1.1 ---------------------------------MAMGATVRAAAADAVVTFLWVLC
ZmSIP1.2 ---------------------------------MAMGEALRAAAADAVVTFLWVLC
ZmSIP2.1 ----------------------------MSPAPSRPRIRPWLVVGDLALAAAWVCA
PpSIP1.1 ------------------------------------MGLARKAVADASITFLWVFA
PpSIP1.2 ------------------------------------MGLARKAVADASITFLWVFA
PpGIP1.1 ----------------------------------MGYNEGQVFAAELLGTTTLCVV
PpHIP1.1 -------------MVKMVTMLNFMKVELGDHKTLRSKEQWRAAAAEFIGTMLFVYL
PpXIP1.1 DEDRGTCKIPEPISAISASKFSSEVLHRAVLRDLQNPEVWRAGVFECVASFAATFV
PpXIP1.2 RVVRNDSVKGHYDGSVVNVPLTRKVKVWIGLHDSRKADVWRAAAVEFVATAGLTFL

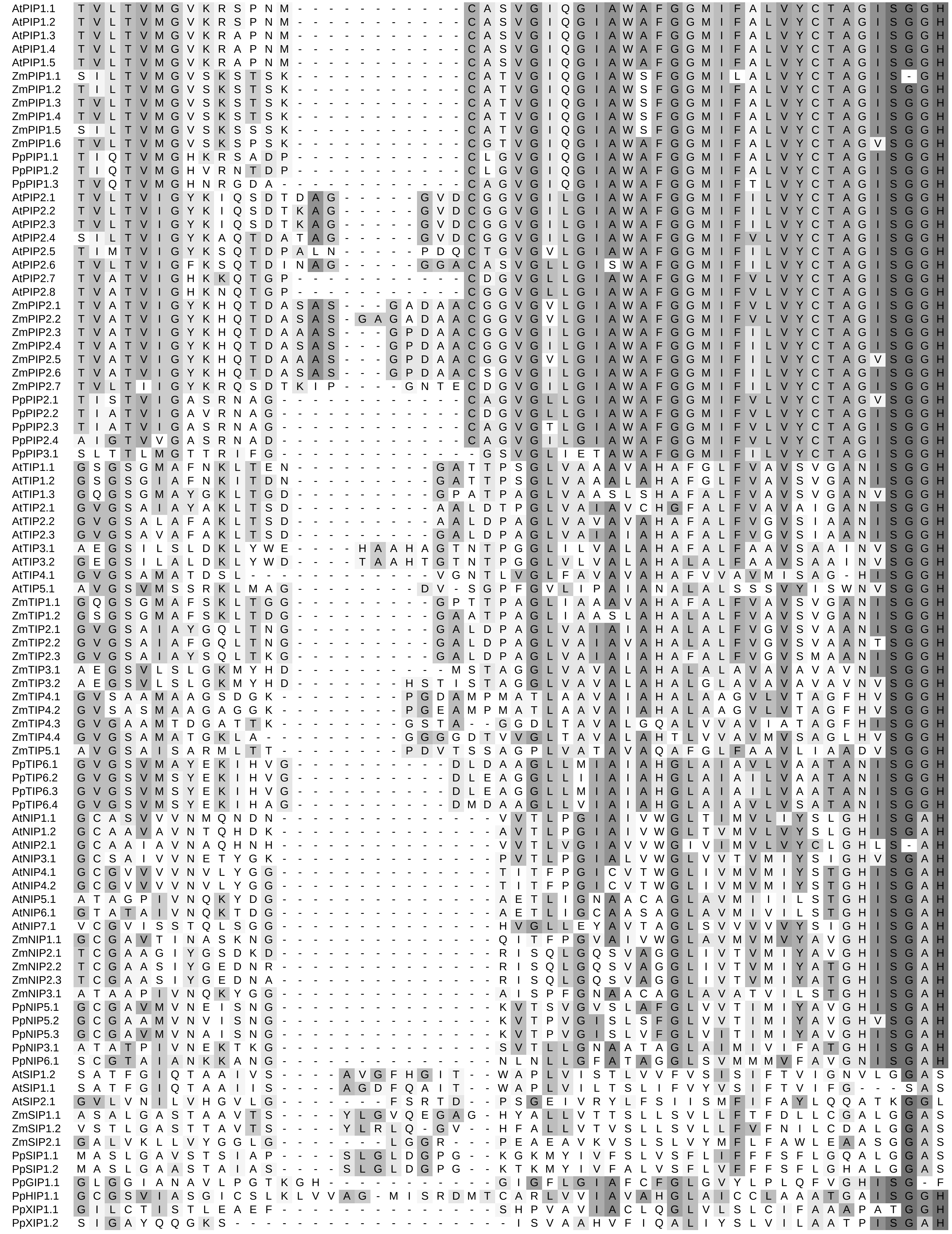
AtPIP1.1
AtPIP1.2
AtPIP1.3
AtPIP1.4
AtPIP1.5
ZmPIP1.1
ZmPIP1.2
ZmPIP1.3
ZmPIP1.4
ZmPIP1.5
ZmPIP1.6
PpPIP1.1
PpPIP1.2
PpPIP1.3
AtPIP2.1
AtPIP2.2
AtPIP2.3
AtPIP2.4
AtPIP2.5
AtPIP2.6
AtPIP2.7
AtPIP2.8
ZmPIP2.1
ZmPIP2.2
ZmPIP2.3
ZmPIP2.4
ZmPIP2.5
ZmPIP2.6
ZmPIP2.7
PpPIP2.1
PpPIP2.2
PpPIP2.3
PpPIP2.4
PpPIP3.1
AtTIP1.1
AtTIP1.2
AtTIP1.3
AtTIP2.1
AtTIP2.2
AtTIP2.3
AtTIP3.1
AtTIP3.2
AtTIP4.1
AtTIP5.1
ZmTIP1.1
ZmTIP1.2
ZmTIP2.1
ZmTIP2.2
ZmTIP2.3
ZmTIP3.1
ZmTIP3.2
ZmTIP4.1
ZmTIP4.2
ZmTIP4.3
ZmTIP4.4
ZmTIP5.1
PpTIP6.1
PpTIP6.2
PpTIP6.3
PpTIP6.4
AtNIP1.1
AtNIP1.2
AtNIP2.1
AtNIP3.1
AtNIP4.1
AtNIP4.2
AtNIP5.1
AtNIP6.1
AtNIP7.1
ZmNIP1.1
ZmNIP2.1
ZmNIP2.2
ZmNIP2.3
ZmNIP3.1
PpNIP5.1
PpNIP5.2
PpNIP5.3
PpNIP3.1
PpNIP6.1
AtSIP1.2
AtSIP1.1
AtSIP2.1
ZmSIP1.1
ZmSIP1.2
ZmSIP2.1
PpSIP1.1
PpSIP1.2
PpGIP1.1
PpHIP1.1
PpXIP1.1
PpXIP1.2

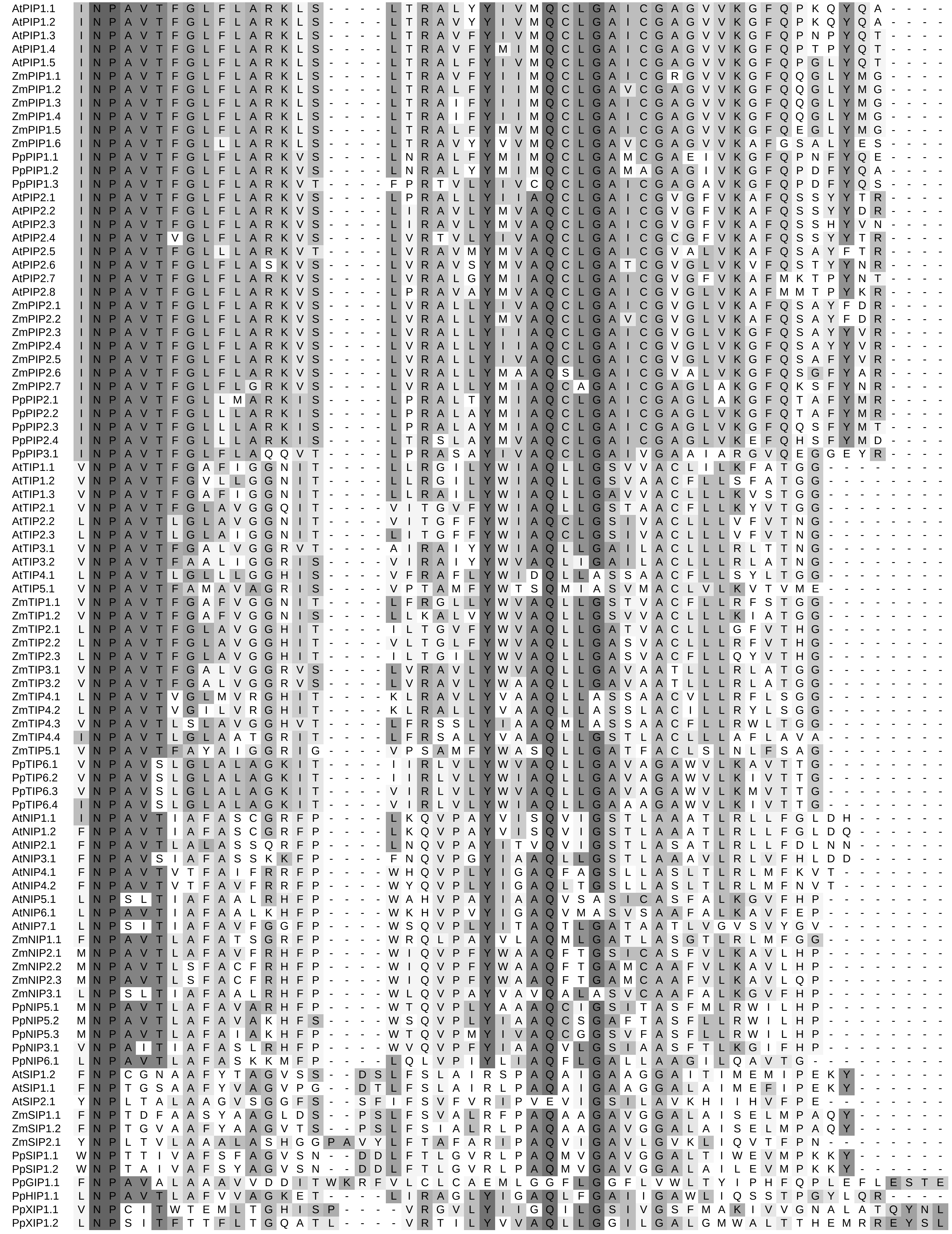

AtPIP1.1 INPAVTFGLFLARKLS----LTRALYYIVMQCLGAICGAGVVKGFQPKQYQA----
AtPIP1.2 INPAVTFGLFLARKLS----LTRAVYYIVMQCLGAICGAGVVKGFQPKQYQA----
AtPIP1.3 INPAVTFGLFLARKLS----LTRAVFYIVMQCLGAICGAGVVKGFQPNPYQT----
AtPIP1.4 INPAVTFGLFLARKLS----LTRAVFYMIMQCLGAICGAGVVKGFQPTPYQT----
AtPIP1.5 INPAVTFGLFLARKLS----LTRALFYIVMQCLGAICGAGVVKGFQPGLYQT----
ZmPIP1.1 INPAVTFGLFLARKLS----LTRAVFYIIMQCLGAICGRGVVKGFQQGLYMG----
ZmPIP1.2 INPAVTFGLFLARKLS----LTRALFYIIMQCLGAVCGAGVVKGFQQGLYMG----
ZmPIP1.3 INPAVTFGLFLARKLS----LTRAIFYIIMQCLGAICGAGVVKGFQQGLYMG----
ZmPIP1.4 INPAVTFGLFLARKLS----LTRAIFYIIMQCLGAICGAGVVKGFQQGLYMG----
ZmPIP1.5 INPAVTFGLFLARKLS----LTRALFYMVMQCLGAICGAGVVKGFQEGLYMG----
ZmPIP1.6 INPAVTFGLLLARKLS----LTRAVYYVVMQCLGAVCGAGVVKAFGSALYES----
PpPIP1.1 INPAVTFGLFLARKVS----LNRALFYMIMQCLGAMCGAEIVKGFQPNFYQE----
PpPIP1.2 INPAVTFGLFLARKVS----LNRALYYMIMQCLGAMAGAGIVKGFQPDFYQA----
PpPIP1.3 INPAVTFGLFLARKVT----FPRTVLYIVCQCLGAICGAGAVKGFQPDFYQS----
AtPIP2.1 INPAVTFGLFLARKVS----LPRALLYIIAQCLGAICGVGFVKAFQSSYYTR----
AtPIP2.2 INPAVTFGLFLARKVS----LIRAVLYMVAQCLGAICGVGFVKAFQSSYYDR----
AtPIP2.3 INPAVTFGLFLARKVS----LIRAVLYMVAQCLGAICGVGFVKAFQSSHYVN----
AtPIP2.4 INPAVTVGLFLARKVS----LVRTVLYIVAQCLGAICGCGFVKAFQSSYYTR----
AtPIP2.5 INPAVTFGLLLARKVT----LVRAVMYMVAQCLGAICGVALVKAFQSAYFTR----
AtPIP2.6 INPAVTFGLFLASKVS----LVRAVSYMVAQCLGATCGVGLVKVFQSTYYNR----
AtPIP2.7 INPAVTFGLFLARKVS----LVRALGYMIAQCLGAICGVGFVKAFMKTPYNT----
AtPIP2.8 INPAVTFGLFLARKVS----LPRAVAYMVAQCLGAICGVGLVKAFMMTPYKR----
ZmPIP2.1 INPAVTFGLFLARKVS----LVRALLYIVAQCLGAICGVGLVKAFQSAYFDR----
ZmPIP2.2 INPAVTFGLFLARKVS----LVRALLYMVAQCLGAVCGVGLVKAFQSAYFDR----
ZmPIP2.3 INPAVTFGLFLARKVS----LVRALLYIIAQCLGAICGVGLVKGFQSAYYVR----
ZmPIP2.4 INPAVTFGLFLARKVS----LVRALLYIIAQCLGAICGVGLVKGFQSAYYVR----
ZmPIP2.5 INPAVTFGLFLARKVS----LVRALLYIVAQCLGAICGVGLVKGFQSAFYVR----
ZmPIP2.6 INPAVTFGLFLARKVS----LVRALLYMAAQSLGAICGVALVKGFQSGFYAR----
ZmPIP2.7 INPAVTFGLFLGRKVS----LVRALLYMIAQCAGAICGAGLAKGFQKSFYNR----
PpPIP2.1 INPAVTFGLLMARKIS----LPRALTYMIAQCLGAICGAGLAKGFQTAFYMR----
PpPIP2.2 INPAVTFGLLLARKIS----LPRALAYMIAQCLGAICGAGLVKGFQTAFYMR----
PpPIP2.3 INPAVTFGLLLARKIS----LPRALAYMIAQCLGAICGAGLVKGFQQSFYMT----
PpPIP2.4 INPAVTFGLLLARKIS----LTRSLAYMVAQCLGAICGAGLVKEFQHSFYMD----
PpPIP3.1 INPAVTFGLFLAQQVT----LPRASAYIVAQCLGAIVGAAIARGVQEGGEYR----
AtTIP1.1 VNPAVTFGAFIGGNIT----LLRGILYWIAQLLGSVVACLILKFATGG--------
AtTIP1.2 VNPAVTFGVLLGGNIT----LLRGILYWIAQLLGSVAACFLLSFATGG--------
AtTIP1.3 VNPAVTFGAFIGGNIT----LLRAILYWIAQLLGAVVACLLLKVSTGG--------
AtTIP2.1 VNPAVTFGLAVGGQIT----VITGVFYWIAQLLGSTAACFLLKYVTGG--------
AtTIP2.2 LNPAVTLGLAVGGNIT----VITGFFYWIAQCLGSIVACLLLVFVTNG--------
AtTIP2.3 LNPAVTLGLAIGGNIT----LITGFFYWIAQCLGSIVACLLLVFVTNG--------
AtTIP3.1 VNPAVTFGALVGGRVT----AIRAIYYWIAQLLGAILACLLLRLTTNG--------
AtTIP3.2 VNPAVTFAALIGGRIS----VIRAIYYWVAQLIGAILACLLLRLATNG--------
AtTIP4.1 LNPAVTLGLLLGGHIS----VFRAFLYWIDQLLASSAACFLLSYLTGG--------
AtTIP5.1 VNPAVTFAMAVAGRIS----VPTAMFYWTSQMIASVMACLVLKVTVME--------
ZmTIP1.1 VNPAVTFGAFVGGNIT----LFRGLLYWVAQLLGSTVACFLLRFSTGG--------
ZmTIP1.2 VNPAVTFGAFVGGNIS----LLKALVYWVAQLLGSVVACLLLKIATGG--------
ZmTIP2.1 LNPAVTFGLAVGGHIT----ILTGVFYWVAQLLGATVACLLLGFVTHG--------
ZmTIP2.2 LNPAVTFGLAVGGHIT----VLTGLFYWVAQLLGASVACLLLRFVTHG--------
ZmTIP2.3 LNPAVTFGLAVGGHIT----ILTGILYWVAQLLGASVACFLLQYVTHG--------
ZmTIP3.1 VNPAVTFGALVGGRVS----LVRAVLYWVAQLLGAVAATLLLRLATGG--------
ZmTIP3.2 VNPAVTFGALVGGRVS----LVRAVLYWAAQLLGAVAATLLLRLATGG--------
ZmTIP4.1 LNPAVTVGLMVRGHIT----KLRAVLYVAAQLLASSAACVLLRFLSGG--------
ZmTIP4.2 LNPAVTVGILVRGHIT----KLRALLYVAAQLLASSLACILLRYLSGG--------
ZmTIP4.3 VNPAVTLSLAVGGHVT----LFRSSLYIAAQMLASSAACFLLRWLTGG--------
ZmTIP4.4 INPAVTLGLAATGRIT----LFRSALYVAAQLLGSTLACLLLAFLAVA--------
ZmTIP5.1 VNPAVTFAYAIGGRIG----VPSAMFYWASQLLGATFACLSLNLFSAG--------
PpTIP6.1 VNPAVSLGLALAGKIT----IIRLVLYWVAQLLGAVAGAWVLKAVTTG--------
PpTIP6.2 VNPAVSLGLALAGKIT----IIRLVLYWIAQLLGAVAGAWVLKIVTTG--------
PpTIP6.3 VNPAVSLGLALAGKIT----VIRLVLYWVAQLLGAVAGAWVLKMVTTG--------
PpTIP6.4 INPAVSLGLALAGKIT----VIRLVLYWIAQLLGAAAGAWVLKIVTTG--------
AtNIP1.1 INPAVTIAFASCGRFP----LKQVPAYVISQVIGSTLAAATLRLLFGLDH------
AtNIP1.2 FNPAVTIAFASCGRFP----LKQVPAYVISQVIGSTLAAATLRLLFGLDQ------
AtNIP2.1 FNPAVTLALASSQRFP----LNQVPAYITVQVIGSTLASATLRLLFDLNN------
AtNIP3.1 FNPAVSIAFASSKKFP----FNQVPGYIAAQLLGSTLAAAVLRLVFHLDD------
AtNIP4.1 FNPAVTVTFAIFRRFP----WHQVPLYIGAQFAGSLLASLTLRLMFKVT-------
AtNIP4.2 FNPAVTVTFAVFRRFP----WYQVPLYIGAQLTGSLLASLTLRLMFNVT-------
AtNIP5.1 LNPSLTIAFAALRHFP----WAHVPAYIAAQVSASICASFALKGVFHP--------
AtNIP6.1 LNPAVTIAFAALKHFP----WKHVPVYIGAQVMASVSAAFALKAVFEP--------
AtNIP7.1 LNPSITIAFAVFGGFP----WSQVPLYITAQTLGATAATLVGVSVYGV--------
ZmNIP1.1 FNPAVTLAFATSGRFP----WRQLPAYVLAQMLGATLASGTLRLMFGG--------
ZmNIP2.1 MNPAVTLAFAVFRHFP----WIQVPFYWAAQFTGSICASFVLKAVLHP--------
ZmNIP2.2 MNPAVTLSFACFRHFP----WIQVPFYWAAQFTGAMCAAFVLKAVLHP--------
ZmNIP2.3 MNPAVTLSFACFRHFP----WIQVPFYWAAQFTGAMCAAFVLKAVLQP--------
ZmNIP3.1 LNPSLTIAFAALRHFP----WLQVPAYVAVQALASVCAAFALKGVFHP--------
PpNIP5.1 MNPAVTLAFAVARHFP----WTQVPLYAAAQCIGSITASFMLRWILHP--------
PpNIP5.2 MNPAVTLAFAVAKHFS----WSQVPLYIAAQCSGAFTASFLLRWILHP--------
PpNIP5.3 MNPAVTLAFAIAKHFP----WTQVPMYIVAQCGGSVFASFLLRWILHP--------
PpNIP3.1 VNPAITIAFASLRHFP----WVQVPFYIAAQVLGSIAASFTLKGIFHP--------
PpNIP6.1 LNPAVTLAFASKKMFP----LQLVPIYLIAQFLGALLAAGILQAVTG---------
AtSIP1.2 FNPCGNAAFYTAGVSS--DSLFSLAIRSPAQAIGAAGGAITIMEMIPEKY------
AtSIP1.1 FNPTGSAAFYVAGVPG--DTLFSLAIRLPAQAIGAAGGALAIMEFIPEKY------
AtSIP2.1 YNPLTALAAGVSGGFS--SFIFSVFVRIPVEVIGSILAVKHIIHVFPE--------
ZmSIP1.1 FNPTDFAASYAAGLDS--PSLFSVALRFPAQAAGAVGGALAISELMPAQY------
ZmSIP1.2 FNPTGVAAFYAAGVTS--PSLFSIALRLPAQAAGAVGGALAISELMPAQY------
ZmSIP2.1 YNPLTVLAAALASHGGPAVYLFTAFARIPAQVIGAVLGVKLIQVTFPN--------
PpSIP1.1 WNPTTIVAFSFAGVSN--DDLFTLGVRLPAQMVGAVGGALTIWEVMPKKY------
PpSIP1.2 WNPTAIVAFSYAGVSN--DDLFTLGVRLPAQMVGAVGGALAILEVMPKKY------
PpGIP1.1 FNPAVALAAAVVDDITWKRFVLCLCAEMLGGFLGGFLVWLTYIPHFQPLEFLESTE
PpHIP1.1 LNPAVTLAFVVAGKET----LIRAGLYIGAQLFGAIIGAWLIQSSTPGYLQR----
PpXIP1.1 VNPCITWTEMLTGHISP----VRGVLYIIGQILGSIVGSFMAKIVVGNALATQYNL
PpXIP1.2 LNPSITFTTFLTGQATL----VRTILYVVAQLLGGILGALGMWALTTHEMRREYSL

```
AtPIP1.1   --------------------------------------------------------
AtPIP1.2   --------------------------------------------------------
AtPIP1.3   --------------------------------------------------------
AtPIP1.4   --------------------------------------------------------
AtPIP1.5   --------------------------------------------------------
ZmPIP1.1   --------------------------------------------------------
ZmPIP1.2   --------------------------------------------------------
ZmPIP1.3   --------------------------------------------------------
ZmPIP1.4   --------------------------------------------------------
ZmPIP1.5   --------------------------------------------------------
ZmPIP1.6   --------------------------------------------------------
PpPIP1.1   --------------------------------------------------------
PpPIP1.2   --------------------------------------------------------
PpPIP1.3   --------------------------------------------------------
AtPIP2.1   --------------------------------------------------------
AtPIP2.2   --------------------------------------------------------
AtPIP2.3   --------------------------------------------------------
AtPIP2.4   --------------------------------------------------------
AtPIP2.5   --------------------------------------------------------
AtPIP2.6   --------------------------------------------------------
AtPIP2.7   --------------------------------------------------------
AtPIP2.8   --------------------------------------------------------
ZmPIP2.1   --------------------------------------------------------
ZmPIP2.2   --------------------------------------------------------
ZmPIP2.3   --------------------------------------------------------
ZmPIP2.4   --------------------------------------------------------
ZmPIP2.5   --------------------------------------------------------
ZmPIP2.6   --------------------------------------------------------
ZmPIP2.7   --------------------------------------------------------
PpPIP2.1   --------------------------------------------------------
PpPIP2.2   --------------------------------------------------------
PpPIP2.3   --------------------------------------------------------
PpPIP2.4   --------------------------------------------------------
PpPIP3.1   --------------------------------------------------------
AtTIP1.1   --------------------------------------------------------
AtTIP1.2   --------------------------------------------------------
AtTIP1.3   --------------------------------------------------------
AtTIP2.1   --------------------------------------------------------
AtTIP2.2   --------------------------------------------------------
AtTIP2.3   --------------------------------------------------------
AtTIP3.1   --------------------------------------------------------
AtTIP3.2   --------------------------------------------------------
AtTIP4.1   --------------------------------------------------------
AtTIP5.1   --------------------------------------------------------
ZmTIP1.1   --------------------------------------------------------
ZmTIP1.2   --------------------------------------------------------
ZmTIP2.1   --------------------------------------------------------
ZmTIP2.2   --------------------------------------------------------
ZmTIP2.3   --------------------------------------------------------
ZmTIP3.1   --------------------------------------------------------
ZmTIP3.2   --------------------------------------------------------
ZmTIP4.1   --------------------------------------------------------
ZmTIP4.2   --------------------------------------------------------
ZmTIP4.3   --------------------------------------------------------
ZmTIP4.4   --------------------------------------------------------
ZmTIP5.1   --------------------------------------------------------
PpTIP6.1   --------------------------------------------------------
PpTIP6.2   --------------------------------------------------------
PpTIP6.3   --------------------------------------------------------
PpTIP6.4   --------------------------------------------------------
AtNIP1.1   --------------------------------------------------------
AtNIP1.2   --------------------------------------------------------
AtNIP2.1   --------------------------------------------------------
AtNIP3.1   --------------------------------------------------------
AtNIP4.1   --------------------------------------------------------
AtNIP4.2   --------------------------------------------------------
AtNIP5.1   --------------------------------------------------------
AtNIP6.1   --------------------------------------------------------
AtNIP7.1   --------------------------------------------------------
ZmNIP1.1   --------------------------------------------------------
ZmNIP2.1   --------------------------------------------------------
ZmNIP2.2   --------------------------------------------------------
ZmNIP2.3   --------------------------------------------------------
ZmNIP3.1   --------------------------------------------------------
PpNIP5.1   --------------------------------------------------------
PpNIP5.2   --------------------------------------------------------
PpNIP5.3   --------------------------------------------------------
PpNIP3.1   --------------------------------------------------------
PpNIP6.1   --------------------------------------------------------
AtSIP1.2   --------------------------------------------------------
AtSIP1.1   --------------------------------------------------------
AtSIP2.1   --------------------------------------------------------
ZmSIP1.1   --------------------------------------------------------
ZmSIP1.2   --------------------------------------------------------
ZmSIP2.1   --------------------------------------------------------
PpSIP1.1   --------------------------------------------------------
PpSIP1.2   --------------------------------------------------------
PpGIP1.1   ESICDCCDLEANGVAIIKQELSEPAHRYASAGTRDNVTTGRVQSEDYEHPMTSFWH
PpHIP1.1   --------------------------------------------------------
PpXIP1.1   --------------------------------------------------------
PpXIP1.2   --------------------------------------------------------
```

```
AtPIP1.1  -----------------------------------------------------LGG
AtPIP1.2  -----------------------------------------------------LGG
AtPIP1.3  -----------------------------------------------------LGG
AtPIP1.4  -----------------------------------------------------LGG
AtPIP1.5  -----------------------------------------------------NGG
ZmPIP1.1  -----------------------------------------------------NGG
ZmPIP1.2  -----------------------------------------------------NGG
ZmPIP1.3  -----------------------------------------------------NGG
ZmPIP1.4  -----------------------------------------------------NGG
ZmPIP1.5  -----------------------------------------------------AGG
ZmPIP1.6  -----------------------------------------------------AGG
PpPIP1.1  -----------------------------------------------------QGG
PpPIP1.2  -----------------------------------------------------QGG
PpPIP1.3  -----------------------------------------------------VGG
AtPIP2.1  -----------------------------------------------------YGG
AtPIP2.2  -----------------------------------------------------YGG
AtPIP2.3  -----------------------------------------------------YGG
AtPIP2.4  -----------------------------------------------------YGG
AtPIP2.5  -----------------------------------------------------YGG
AtPIP2.6  -----------------------------------------------------YGG
AtPIP2.7  -----------------------------------------------------LGG
AtPIP2.8  -----------------------------------------------------LGG
ZmPIP2.1  -----------------------------------------------------YGG
ZmPIP2.2  -----------------------------------------------------YGG
ZmPIP2.3  -----------------------------------------------------YGG
ZmPIP2.4  -----------------------------------------------------YGG
ZmPIP2.5  -----------------------------------------------------YGG
ZmPIP2.6  -----------------------------------------------------YGG
ZmPIP2.7  -----------------------------------------------------YGG
PpPIP2.1  -----------------------------------------------------YGG
PpPIP2.2  -----------------------------------------------------YGG
PpPIP2.3  -----------------------------------------------------YGG
PpPIP2.4  -----------------------------------------------------HGG
PpPIP3.1  ---------------------------------------------------SFASN
AtTIP1.1  -----------------------------------------------------LAV
AtTIP1.2  -----------------------------------------------------EPI
AtTIP1.3  -----------------------------------------------------MET
AtTIP2.1  -----------------------------------------------------LAV
AtTIP2.2  -----------------------------------------------------ESV
AtTIP2.3  -----------------------------------------------------KSV
AtTIP3.1  -----------------------------------------------------MRP
AtTIP3.2  -----------------------------------------------------LRP
AtTIP4.1  -----------------------------------------------------MGT
AtTIP5.1  -----------------------------------------------------QHV
ZmTIP1.1  -----------------------------------------------------QAT
ZmTIP1.2  -----------------------------------------------------AAL
ZmTIP2.1  -----------------------------------------------------KAI
ZmTIP2.2  -----------------------------------------------------KAI
ZmTIP2.3  -----------------------------------------------------QAI
ZmTIP3.1  -----------------------------------------------------MRP
ZmTIP3.2  -----------------------------------------------------ARP
ZmTIP4.1  -----------------------------------------------------MVT
ZmTIP4.2  -----------------------------------------------------MVT
ZmTIP4.3  -----------------------------------------------------LAT
ZmTIP4.4  ----------------------------------------------------DSGV
ZmTIP5.1  -----------------------------------------------------EEV
PpTIP6.1  -----------------------------------------------------EDV
PpTIP6.2  -----------------------------------------------------EDL
PpTIP6.3  -----------------------------------------------------EDV
PpTIP6.4  -----------------------------------------------------EDV
AtNIP1.1  ------------------------------------------------DVCSGKHD
AtNIP1.2  ------------------------------------------------DVCSGKHD
AtNIP2.1  ------------------------------------------------DVCSKKHD
AtNIP3.1  ------------------------------------------------DVCSLKGD
AtNIP4.1  ------------------------------------------------------PE
AtNIP4.2  ------------------------------------------------------PK
AtNIP5.1  ------------------------------------------------------FM
AtNIP6.1  ------------------------------------------------------TM
AtNIP7.1  ------------------------------------------------------NA
ZmNIP1.1  -----------------------------------------------------RHE
ZmNIP2.1  ------------------------------------------------------IA
ZmNIP2.2  ------------------------------------------------------IA
ZmNIP2.3  ------------------------------------------------------IA
ZmNIP3.1  ------------------------------------------------------FL
PpNIP5.1  ------------------------------------------------------AA
PpNIP5.2  ------------------------------------------------------AA
PpNIP5.3  ------------------------------------------------------AA
PpNIP3.1  ------------------------------------------------------YM
PpNIP6.1  ------------------------------------------------------DT
AtSIP1.2  -------------------------------------------------------K
AtSIP1.1  -------------------------------------------------------K
AtSIP2.1  --------------------------------------------------------
ZmSIP1.1  -------------------------------------------------------K
ZmSIP1.2  -------------------------------------------------------R
ZmSIP2.1  --------------------------------------------------------
PpSIP1.1  -------------------------------------------------------K
PpSIP1.2  -------------------------------------------------------K
PpGIP1.1  ELLRKIMGCGSHSSELKKESDKGSMDRLHQARAKSMKSACGPSVKQRIAEDQEVKL
PpHIP1.1  ------------------------------------------------------GL
PpXIP1.1  -------------------------------------------------GGCYLQS
PpXIP1.2  ------------------------------------------GGCLLQ-----KLP
```

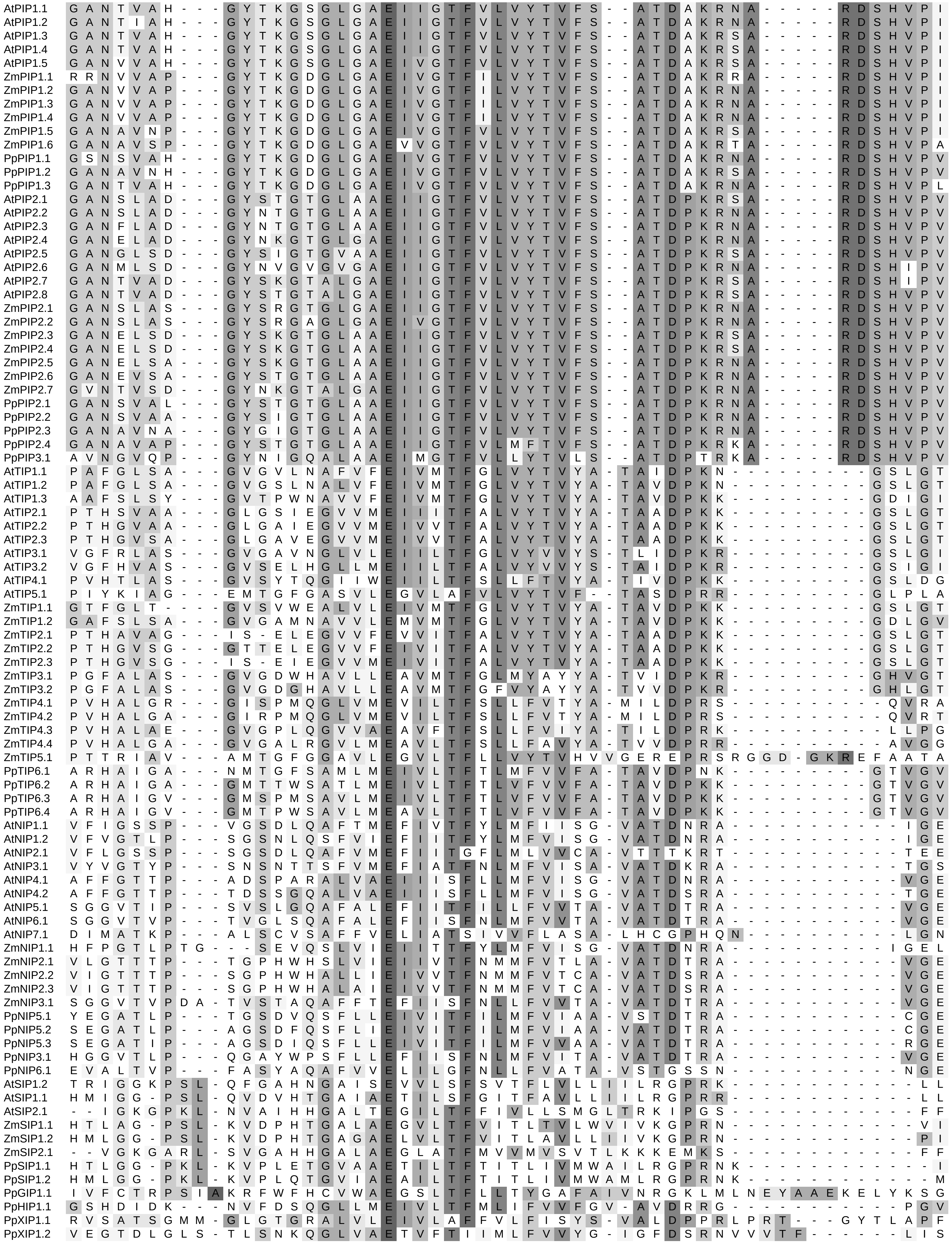
AtPIP1.1
AtPIP1.2
AtPIP1.3
AtPIP1.4
AtPIP1.5
ZmPIP1.1
ZmPIP1.2
ZmPIP1.3
ZmPIP1.4
ZmPIP1.5
ZmPIP1.6
PpPIP1.1
PpPIP1.2
PpPIP1.3
AtPIP2.1
AtPIP2.2
AtPIP2.3
AtPIP2.4
AtPIP2.5
AtPIP2.6
AtPIP2.7
AtPIP2.8
ZmPIP2.1
ZmPIP2.2
ZmPIP2.3
ZmPIP2.4
ZmPIP2.5
ZmPIP2.6
ZmPIP2.7
PpPIP2.1
PpPIP2.2
PpPIP2.3
PpPIP2.4
PpPIP3.1
AtTIP1.1
AtTIP1.2
AtTIP1.3
AtTIP2.1
AtTIP2.2
AtTIP2.3
AtTIP3.1
AtTIP3.2
AtTIP4.1
AtTIP5.1
ZmTIP1.1
ZmTIP1.2
ZmTIP2.1
ZmTIP2.2
ZmTIP2.3
ZmTIP3.1
ZmTIP3.2
ZmTIP4.1
ZmTIP4.2
ZmTIP4.3
ZmTIP4.4
ZmTIP5.1
PpTIP6.1
PpTIP6.2
PpTIP6.3
PpTIP6.4
AtNIP1.1
AtNIP1.2
AtNIP2.1
AtNIP3.1
AtNIP4.1
AtNIP4.2
AtNIP5.1
AtNIP6.1
AtNIP7.1
ZmNIP1.1
ZmNIP2.1
ZmNIP2.2
ZmNIP2.3
ZmNIP3.1
PpNIP5.1
PpNIP5.2
PpNIP5.3
PpNIP3.1
PpNIP6.1
AtSIP1.2
AtSIP1.1
AtSIP2.1
ZmSIP1.1
ZmSIP1.2
ZmSIP2.1
PpSIP1.1
PpSIP1.2
PpGIP1.1
PpHIP1.1
PpXIP1.1
PpXIP1.2

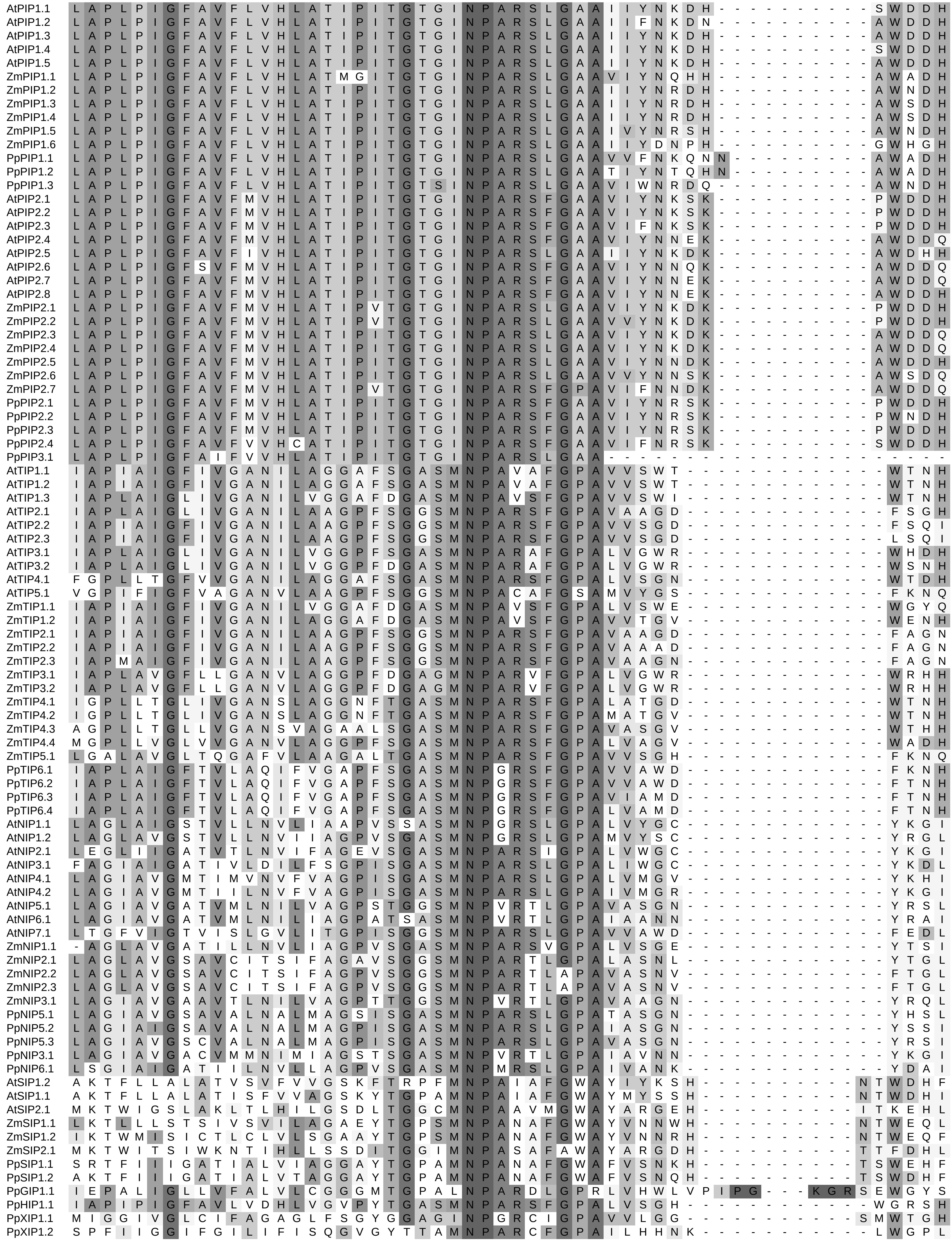

AtPIP1.1 LAPLPIGFAVFLVHLATIPITGTGINPARSLGAAIIYNKDH---------SWDDH
AtPIP1.2 LAPLPIGFAVFLVHLATIPITGTGINPARSLGAAIIFNKDN---------AWDDH
AtPIP1.3 LAPLPIGFAVFLVHLATIPITGTGINPARSLGAAIIYNKDH---------AWDDH
AtPIP1.4 LAPLPIGFAVFLVHLATIPITGTGINPARSLGAAIIYNKDH---------SWDDH
AtPIP1.5 LAPLPIGFAVFLVHLATIPITGTGINPARSLGAAIIYNKDH---------AWDDH
ZmPIP1.1 LAPLPIGFAVFLVHLATMGITGTGINPARSLGAAVIYNQHH---------AWADH
ZmPIP1.2 LAPLPIGFAVFLVHLATIPITGTGINPARSLGAAIIYNRDH---------AWNDH
ZmPIP1.3 LAPLPIGFAVFLVHLATIPITGTGINPARSLGAAIIYNRDH---------AWSDH
ZmPIP1.4 LAPLPIGFAVFLVHLATIPITGTGINPARSLGAAIIYNRDH---------AWSDH
ZmPIP1.5 LAPLPIGFAVFLVHLATIPITGTGINPARSLGAAIVYNRSH---------AWNDH
ZmPIP1.6 LAPLPIGFAVFLVHLATIPITGTGINPARSLGAAIIYDNPH---------GWHGH
PpPIP1.1 LAPLPIGFAVFLVHLATIPITGTGINPARSLGAAVVFNKQNN--------AWADH
PpPIP1.2 LAPLPIGFAVFLVHLATIPITGTGINPARSLGAATIYNTQHN--------AWADH
PpPIP1.3 LAPLPIGFAVFLVHLATIPITGTSINPARSLGAAVIWNRDQ---------AWNDH
AtPIP2.1 LAPLPIGFAVFMVHLATIPITGTGINPARSFGAAVIYNKSK---------PWDDH
AtPIP2.2 LAPLPIGFAVFMVHLATIPITGTGINPARSFGAAVIYNKSK---------PWDDH
AtPIP2.3 LAPLPIGFAVFMVHLATIPITGTGINPARSFGAAVIFNKSK---------PWDDH
AtPIP2.4 LAPLPIGFAVFMVHLATIPITGTGINPARSFGAAVIYNNEK---------AWDDQ
AtPIP2.5 LAPLPIGFAVFIVHLATIPITGTGINPARSLGAAIIYNKDK---------AWDHH
AtPIP2.6 LAPLPIGFSVFMVHLATIPITGTGINPARSFGAAVIYNNQK---------AWDDQ
AtPIP2.7 LAPLPIGFAVFMVHLATIPITGTGINPARSFGAAVIYNNEK---------AWDDQ
AtPIP2.8 LAPLPIGFAVFMVHLATIPITGTGINPARSFGAAVIYNNEK---------AWDDH
ZmPIP2.1 LAPLPIGFAVFMVHLATIPVTGTGINPARSLGAAVIYNKDK---------PWDDH
ZmPIP2.2 LAPLPIGFAVFMVHLATIPVTGTGINPARSLGAAVVYNKDK---------PWDDH
ZmPIP2.3 LAPLPIGFAVFMVHLATIPITGTGINPARSLGAAVIYNKDK---------AWDDQ
ZmPIP2.4 LAPLPIGFAVFMVHLATIPITGTGINPARSLGAAVIYNKDK---------AWDDQ
ZmPIP2.5 LAPLPIGFAVFMVHLATIPITGTGINPARSLGAAVIYNNDK---------AWDDH
ZmPIP2.6 LAPLPIGFAVFMVHLATIPITGTGINPARSLGAAVVYNNSK---------AWSDQ
ZmPIP2.7 LAPLPIGFAVFMVHLATIPVTGTGINPARSFGPAVIFNNDK---------AWDDQ
PpPIP2.1 LAPLPIGFAVFMVHLATIPITGTGINPARSFGAAVIYNRSK---------PWDDH
PpPIP2.2 LAPLPIGFAVFMVHLATIPITGTGINPARSFGAAVIYNRSK---------PWNDH
PpPIP2.3 LAPLPIGFAVFMVHLATIPITGTGINPARSFGAAVIYNRSK---------PWDDH
PpPIP2.4 LAPLPIGFAVFVVHCATIPITGTGINPARSFGAAVIFNRSK---------SWDDH
PpPIP3.1 LAPLPIGFAIFVVHLATIPITGTGINPARSLGAA---------------------
AtTIP1.1 IAPIAIGFIVGANILAGGAFSGASMNPAVAFGPAVVSWT-----------WTNH
AtTIP1.2 IAPIAIGFIVGANILAGGAFSGASMNPAVAFGPAVVSWT-----------WTNH
AtTIP1.3 IAPLAIGLIVGANILVGGAFDGASMNPAVSFGPAVVSWI-----------WTNH
AtTIP2.1 IAPLAIGLIVGANILAAGPFSGGSMNPARSFGPAVAAGD-----------FSGH
AtTIP2.2 IAPIAIGFIVGANILAAGPFSGGSMNPARSFGPAVVSGD-----------FSQI
AtTIP2.3 IAPIAIGFIVGANILAAGPFSGGSMNPARSFGPAVVSGD-----------LSQI
AtTIP3.1 IAPLAIGLIVGANILVGGPFSGASMNPARAFGPALVGWR-----------WHDH
AtTIP3.2 IAPLAIGLIVGANILVGGPFDGASMNPARAFGPALVGWR-----------WSNH
AtTIP4.1 FGPLLTGFVVGANILAGGAFSGASMNPARSFGPALVSGN-----------WTDH
AtTIP5.1 VGPIFIGFVAGANVLAAGPFSGGSMNPACAFGSAMVYGS-----------FKNQ
ZmTIP1.1 IAPIAIGFIVGANILVGGAFDGASMNPAVSFGPALVSWE-----------WGYQ
ZmTIP1.2 IAPIAIGFIVGANILAGGAFDGASMNPAVSFGPAVVTGV-----------WENH
ZmTIP2.1 IAPIAIGFIVGANILAAGPFSGGSMNPARSFGPAVAAGD-----------FAGN
ZmTIP2.2 IAPIAIGFIVGANILAAGPFSGGSMNPARSFGPAVAAAD-----------FAGN
ZmTIP2.3 IAPMAIGFIVGANILAAGPFSGGSMNPARSFGPAVAAGN-----------FAGN
ZmTIP3.1 IAPLAVGFLLGANVLAGGPFDGAGMNPARVFGPALVGWR-----------WRHH
ZmTIP3.2 IAPLAVGFLLGANVLAGGPFDGAGMNPARVFGPALVGWR-----------WRHH
ZmTIP4.1 IGPLLTGLIVGANSLAGGNFTGASMNPARSFGPALATGD-----------WTNH
ZmTIP4.2 IGPLLTGLIVGANSLAGGNFTGASMNPARSFGPAMATGV-----------WTNH
ZmTIP4.3 AGPLLTGLLVGANSVAGAALSGASMNPARSFGPAVASGV-----------WTHH
ZmTIP4.4 MGPLLVGLVVGANVLAGGPFSGASMNPARSFGPALVAGV-----------WADH
ZmTIP5.1 LGALAVGLTQGAFVLAAGALTGASMNPARSFGPAVVSGH-----------FKNQ
PpTIP6.1 IAPLAIGFTVLAQIFVGAPFSGASMNPGRSFGPAVVAWD-----------FKNH
PpTIP6.2 IAPLAIGFTVLAQIFVGAPFSGASMNPGRSFGPAVVAWD-----------FTNH
PpTIP6.3 IAPLAIGFTVLAQIFVGAPFSGASMNPGRSFGPAVIAMD-----------FTNH
PpTIP6.4 IAPLAIGFTVLAQIFVGAPFSGASMNPGRSFGPALVAMD-----------FTNH
AtNIP1.1 LAGLAIGSTVLLNVLIAAPVSSASMNPGRSLGPALVYGC-----------YKGI
AtNIP1.2 LAGLAVGSTVLLNVIIAGPVSGASMNPGRSLGPAMVYSC-----------YRGL
AtNIP2.1 LEGLIIGATVTLNVIFAGEVSGASMNPARSIGPALVWGC-----------YKGI
AtNIP3.1 FAGIAIGATIVLDILFSGPISGASMNPARSLGPALIWGC-----------YKDL
AtNIP4.1 LAGIAVGMTIMVNVFVAGPISGASMNPARSLGPALVMGV-----------YKHI
AtNIP4.2 LAGIAVGMTIILNVFVAGPISGASMNPARSLGPAIVMGR-----------YKGI
AtNIP5.1 LAGIAVGATVMLNILVAGPSTGGSMNPVRTLGPAVASGN-----------YRSL
AtNIP6.1 LAGIAVGATVMLNLIIAGPATSASMNPVRTLGPAIAANN-----------YRAI
AtNIP7.1 LTGFVIGTVISLGVLITGPISGGSMNPARSLGPAVVAWD-----------FEDL
ZmNIP1.1 -AGLAVGATILLNVLIAGPVSGASMNPARSVGPALVSGE-----------YTSI
ZmNIP2.1 LAGLAVGSAVCITSIFAGAVSGGSMNPARTLGPALASNL-----------YTGL
ZmNIP2.2 LAGLAVGSAVCITSIFAGPVSGGSMNPARTLAPAVASNV-----------FTGL
ZmNIP2.3 LAGLAVGSAVCITSIFAGPVSGGSMNPARTLAPAVASNV-----------FTGL
ZmNIP3.1 LAGIAVGAAVTLNILVAGPTTGGSMNPVRTLGPAVAAGN-----------YRQL
PpNIP5.1 LAGIAVGSAVALNALMAGSISGASMNPARSLGPATASGN-----------YHSL
PpNIP5.2 LAGIAIGSAVALNALMAGPISGASMNPARSLGPAIASGN-----------YSSI
PpNIP5.3 LAGIAVGSCVALNALMAGPISGASMNPARSLGPAVASGN-----------YRSI
PpNIP3.1 LAGIAVGACVMMNIMIAGSTSGASMNPVRTLGPAIAVNN-----------YKGI
PpNIP6.1 LSGIAIGATIILNVLLAGPVSGASMNPMRSLGPAIVANK-----------YDAI
AtSIP1.2 AKTFLLALATVSVFVVGSKFTRPFMNPAIAFGWAYIYKSH   NTWDHF
AtSIP1.1 AKTFLLALATISFVVAGSKYTGPAMNPAIAFGWAYMYSSH   NTWDHI
AtSIP2.1 MKTWIGSLAKLTLHILGSDLTGGCMNPAAVMGWAYARGEH   ITKEHL
ZmSIP1.1 LKTLLLSTSIVSVILAGAEYTGPSMNPANAFGWAYVNNWH   NTWEQL
ZmSIP1.2 IKTWMISICTLCLVLSGAAYTGPSMNPANAFGWAYVNNRH   NTWEQF
ZmSIP2.1 MKTWITSIWKNTIHLLSSDITGGIMNPASAFAWAYARGDH   TTFDHL
PpSIP1.1 SRTFIIIGATIALVIAGGAYTGPAMNPANAFGWAFVSNKH   TSWEHF
PpSIP1.2 AKTFIIIGATIALVTAGGAYTGPAMNPANAFGWAFVSNQH   TSWDHF
PpGIP1.1 IEPALIGLLVFALVLCGGGMTGPALNPARDLGPRLVHWLVPIPG---KGRSEWGYS
PpHIP1.1 IAPIPIGFAVLVDHLVGVPYTGASMNPARSFGPALVSGH-----------WGRSH
PpXIP1.1 MIGGIVGLCIFAGAGLFSGYGGAGIMNPGRCIGPAVVLGG---------SMWTGH
PpXIP1.2 SPFIIGGIFGILIFISQGVGYTTAMNPARCFGPAILHHNK----------LWGPL

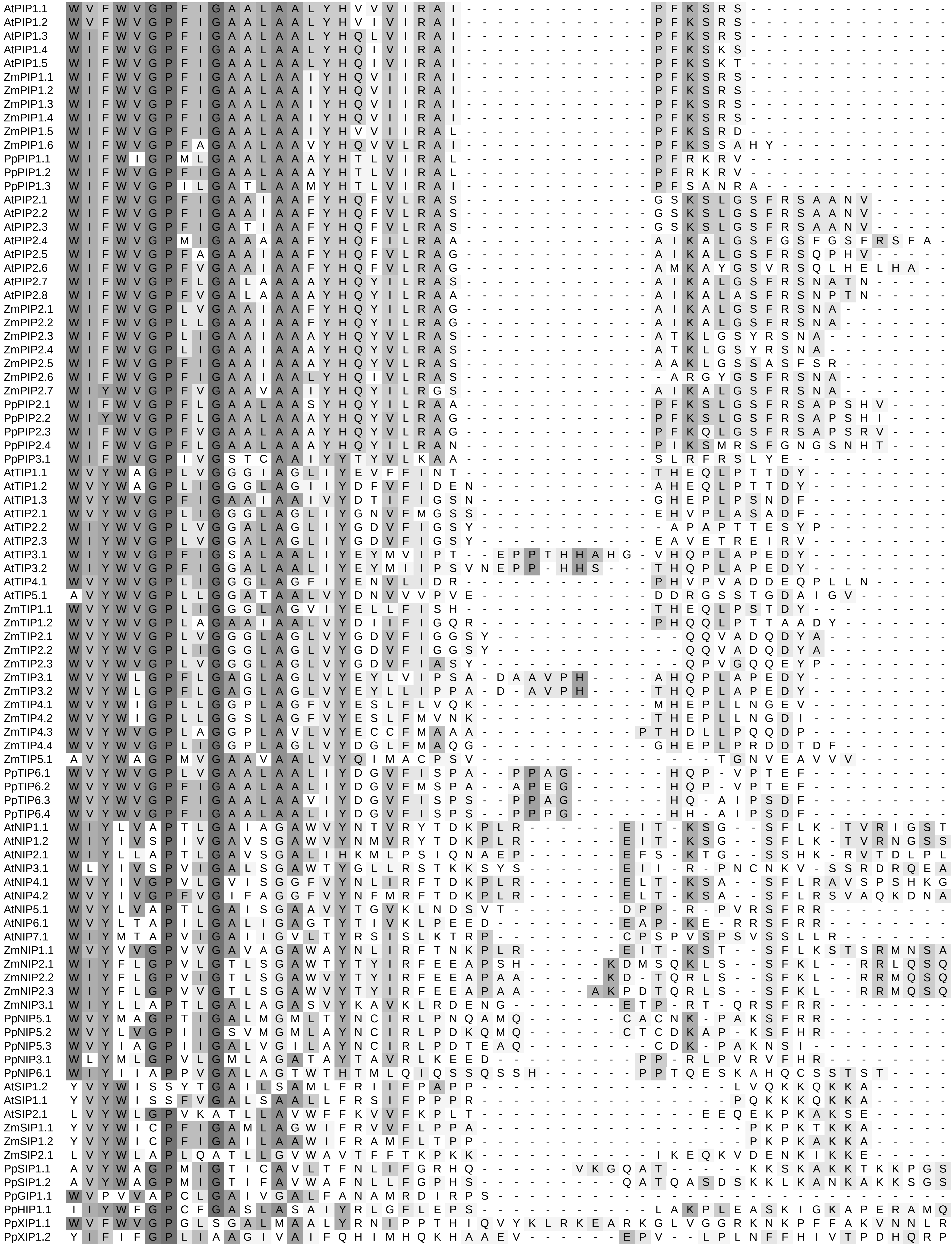

AtPIP1.1 WVFWVGPFIGAALAALYHVVVIRAI------------PFKSRS-------------
AtPIP1.2 WVFWVGPFIGAALAALYHVIVIRAI------------PFKSRS-------------
AtPIP1.3 WIFWVGPFIGAALAALYHQLVIRAI------------PFKSRS-------------
AtPIP1.4 WIFWVGPFIGAALAALYHQIVIRAI------------PFKSKS-------------
AtPIP1.5 WIFWVGPFIGAALAALYHQIVIRAI------------PFKSKT-------------
ZmPIP1.1 WIFWVGPFIGAALAAIYHQVIIRAI------------PFKSRS-------------
ZmPIP1.2 WIFWVGPFIGAALAAIYHQVIIRAI------------PFKSRS-------------
ZmPIP1.3 WIFWVGPFIGAALAAIYHQVIIRAI------------PFKSRS-------------
ZmPIP1.4 WIFWVGPFIGAALAAIYHQVIIRAI------------PFKSRS-------------
ZmPIP1.5 WIFWVGPFIGAALAAIYHVVIIRAL------------PFKSRD-------------
ZmPIP1.6 WIFWVGPFAGAALAAVYHQVVLRAI------------PFKSSAHY-----------
PpPIP1.1 WIFWIGPMLGAALAAAYHTLVIRAL------------PFRKRV-------------
PpPIP1.2 WIFWVGPFIGAALAAAYHTLVIRAL------------PFRKRV-------------
PpPIP1.3 WIFWVGPILGATLAAMYHTLVIRAI------------PFSANRA------------
AtPIP2.1 WIFWVGPFIGAAIAAFYHQFVLRAS------------GSKSLGSFRSAANV-----
AtPIP2.2 WIFWVGPFIGAAIAAFYHQFVLRAS------------GSKSLGSFRSAANV-----
AtPIP2.3 WIFWVGPFIGATIAAFYHQFVLRAS------------GSKSLGSFRSAANV-----
AtPIP2.4 WIFWVGPMIGAAAAAFYHQFILRAA------------AIKALGSFGSFGSFRSFA-
AtPIP2.5 WIFWVGPFAGAAIAAFYHQFVLRAG------------AIKALGSFRSQPHV-----
AtPIP2.6 WIFWVGPFVGAAIAAFYHQFVLRAG------------AMKAYGSVRSQLHELHA--
AtPIP2.7 WIFWVGPFLGALAAAAYHQYILRAS------------AIKALGSFRSNATN-----
AtPIP2.8 WIFWVGPFVGALAAAAYHQYILRAA------------AIKALASFRSNPTN-----
ZmPIP2.1 WIFWVGPLVGAAIAAFYHQYILRAG------------AIKALGSFRSNA-------
ZmPIP2.2 WIFWVGPLLGAAIAAFYHQYILRAG------------AIKALGSFRSNA-------
ZmPIP2.3 WIFWVGPLIGAAIAAAYHQYVLRAS------------ATKLGSYRSNA--------
ZmPIP2.4 WIFWVGPLIGAAIAAAYHQYVLRAS------------ATKLGSYRSNA--------
ZmPIP2.5 WIFWVGPFIGAAIAAAYHQYVLRAS------------AAKLGSSASFSR-------
ZmPIP2.6 WIFWVGPFIGAAIAALYHQIVLRAS-------------ARGYGSFRSNA-------
ZmPIP2.7 WIYWVGPFVGAAVAAIYHQYILRGS------------AIKALGSFRSNA-------
PpPIP2.1 WIFWVGPFLGAALAASYHQYILRAA------------PFKSLGSFRSAPSHV----
PpPIP2.2 WIYWVGPFLGAALAAAYHQYVLRAG------------PFKSLGSFRSAPSHI----
PpPIP2.3 WIFWVGPFVGAALAAAYHQYVLRAG------------PFKQLGSFRSAPSRV----
PpPIP2.4 WIFWVGPFLGAALAAAYHQYILRAN------------PIKSMRSFGNGSNHT----
PpPIP3.1 WIFWVGPIVGSTCAAIYYTYVLKAA------------SLRFRSLYE----------
AtTIP1.1 WVYWAGPLVGGGIAGLIYEVFFINT------------THEQLPTTDY---------
AtTIP1.2 WVYWAGPLIGGGLAGIIYDFVFIDEN-----------AHEQLPTTDY---------
AtTIP1.3 WVYWVGPFIGAAIAAIVYDTIFIGSN-----------GHEPLPSNDF---------
AtTIP2.1 WVYWVGPLIGGGLAGLIYGNVFMGSSS----------EHVPLASADF---------
AtTIP2.2 WIYWVGPLVGGALAGLIYGDVFIGSY------------APAPTTESYP--------
AtTIP2.3 WIYWVGPLVGGALAGLIYGDVFIGSY-----------EAVETREIRV---------
AtTIP3.1 WIYWVGPFIGSALAALIYEYMVIPT--EPPTHHAHG-VHQPLAPEDY---------
AtTIP3.2 WIYWVGPFIGGALAALIYEYMIIPSVNEPP-HHS---THQPLAPEDY---------
AtTIP4.1 WVYWVGPLIGGGLAGFIYENVLIDR------------PHVPVADDEQPLLN-----
AtTIP5.1 AVYWVGPLLGGATAALVYDNVVVPVE-----------DDRGSSTGDAIGV------
ZmTIP1.1 WVYWVGPLIGGGLAGVIYELLFISH------------THEQLPSTDY---------
ZmTIP1.2 WVYWVGPLAGAAIAALVYDIIFIGQR-----------PHQQLPTTAADY-------
ZmTIP2.1 WVYWVGPLVGGGLAGLVYGDVFIGGSY------------QQVADQDYA--------
ZmTIP2.2 WVYWVGPLIGGGLAGLVYGDVFIGGSY------------QQVADQDYA--------
ZmTIP2.3 WVYWVGPLVGGGLAGLVYGDVFIASY-------------QPVGQQEYP--------
ZmTIP3.1 WVYWLGPFLGAGLAGLVYEYLVIPSA-DAAVPH----AHQPLAPEDY---------
ZmTIP3.2 WVYWLGPFLGAGLAGLVYEYLLIPPA-D-AVPH----THQPLAPEDY---------
ZmTIP4.1 WVYWIGPLLGGPLAGFVYESLFLVQK-----------MHEPLLNGEV---------
ZmTIP4.2 WVYWIGPLLGGSLAGFVYESLFMVNK-----------THEPLLNGDI---------
ZmTIP4.3 WVYWVGPLAGGPLAVLVYECCFMAAA----------PTHDLLPQQDP---------
ZmTIP4.4 WVYWVGPLIGGPLAGLVYDGLFMAQG-----------GHEPLPRDDTDF-------
ZmTIP5.1 AVYWAGPMVGAAVAALVYQIMACPSV---------------TGNVEAVVV------
PpTIP6.1 WVYWVGPLVGAALAALIYDGVFISPA--PPAG------HQP-VPTEF---------
PpTIP6.2 WVYWVGPFIGAALAALIYDGVFMSPA--APEG------HQP-VPTEF---------
PpTIP6.3 WVYWVGPFIGAALAAVIYDGVFISPS--PPAG------HQ-AIPSDF---------
PpTIP6.4 WVYWVGPFIGAALAALIYDGVFISPS--PPPG------HH-AIPSDF---------
AtNIP1.1 WIYLVAPTLGAIAGAWVYNTVRYTDKPLR------EIT-KSG--SFLK-TVRIGST
AtNIP1.2 WIYIVSPIVGAVSGAWVYNMVRYTDKPLR------EIT-KSG--SFLK-TVRNGSS
AtNIP2.1 WIYLLAPTLGAVSGALIHKMLPSIQNAEP------EFS-KTG--SSHK-RVTDLPL
AtNIP3.1 WLYIVSPVIGALSGAWTYGLLRSTKKSYS------EII-R-PNCNKV-SSRDRQEA
AtNIP4.1 WVYIVGPVLGVISGGFVYNLIRFTDKPLR------ELT-KSA--SFLRAVSPSHKG
AtNIP4.2 WVYIVGPFVGIFAGGFVYNFMRFTDKPLR------ELT-KSA--SFLRSVAQKDNA
AtNIP5.1 WVYLVAPTLGAISGAAVYTGVKLNDSVT-------DPP-R-PVRSFRR--------
AtNIP6.1 WVYLTAPILGALIGAGTYTIVKLPEED--------EAP-KE-RRSFRR--------
AtNIP7.1 WIYMTAPVIGAIIGVLTYRSISLKTRP--------CPSPVSPSVSSLLR-------
ZmNIP1.1 WVYVVGPVVGAVAGAWAYNLIRFTNKPLR------EIT-KST--SFLKSTSRMNSA
ZmNIP2.1 WIYFLGPVLGTLSGAWTYTYIRFEEAPSH-----KDMSQKLS--SFKL--RRLQSQ
ZmNIP2.2 WIYFLGPVIGTLSGAWVYTYIRFEEAPAA-----KD-TQRLS--SFKL--RRMQSQ
ZmNIP2.3 WIYFLGPVVGTLSGAWVYTYIRFEEAPAA----AKPDTQRLS--SFKL--RRMQSQ
ZmNIP3.1 WIYLLAPTLGALAGASVYKAVKLRDENG-------ETP-RT-QRSFRR--------
PpNIP5.1 WVYMAGPTIGALMGMLTYNCIRLPNQAMQ------CACNK-PAKSFRR--------
PpNIP5.2 WVYLVGPIIGSVMGMLAYNCIRLPDKQMQ------CTCDKAP-KSFHR--------
PpNIP5.3 WVYIAGPIIGALVGILAYNCIRLPDTEAQ--------CDK-PAKNSI---------
PpNIP3.1 WLYMLGPVLGMLAGATAYTAVRLKEED---------PP-RLPVRVFHR--------
PpNIP6.1 WIYIIAPPVGALAGTWTHTMLQIQSSQSSH------PPTQESKAHQCSSTST----
AtSIP1.2 YVYWISSYTGAILSAMLFRIIFPAPP----------------LVQKKQKKA-----
AtSIP1.1 YVYWISSFVGALSAALLFRSIFPPPR----------------PQKKKQKKA-----
AtSIP2.1 LVYWLGPVKATLLAVWFFKVVFKPLT--------------EEQEKPKAKSE-----
ZmSIP1.1 YVYWICPFIGAMLAGWIFRVVFLPPA-----------------PKPKTKKA-----
ZmSIP1.2 YVYWICPFIGAILAAWIFRAMFLTPP-----------------PKPKAKKA-----
ZmSIP2.1 LVYWLAPLQATLLGVWAVTFFTKPKK-----------IKEQKVDENKIKKE-----
PpSIP1.1 AVYWAGPMIGTICAVLTFNLIFGRHQ------VKGQAT-----KKSKAKKTKKPGS
PpSIP1.2 AVYWAGPMIGTIFAVWAFNLLFGPHS---------QATQASDSKKLKANKAKKSGS
PpGIP1.1 WVPVVAPCLGAIVGALFANAMRDIRPS-----------------------------
PpHIP1.1 IIYWFGPCFGASLASAIYRLGFLEPS-----------LAKPLEASKIGKAPERAMQ
PpXIP1.1 WVFWVGPGLSGALMAALYRNIPPTHIQVYKLRKEARKGLVGGRKNKPFFAKVNNLR
PpXIP1.2 YIFIFGPLIAAGIVAIFQHIMHQKHAAEV------EPV--LPLNFFHIVTPDHQRP

AtPIP1.1	--
AtPIP1.2	--
AtPIP1.3	--
AtPIP1.4	--
AtPIP1.5	--
ZmPIP1.1	--
ZmPIP1.2	--
ZmPIP1.3	--
ZmPIP1.4	--
ZmPIP1.5	--
ZmPIP1.6	--
PpPIP1.1	--
PpPIP1.2	--
PpPIP1.3	--
AtPIP2.1	--
AtPIP2.2	--
AtPIP2.3	--
AtPIP2.4	--
AtPIP2.5	--
AtPIP2.6	--
AtPIP2.7	--
AtPIP2.8	--
ZmPIP2.1	--
ZmPIP2.2	--
ZmPIP2.3	--
ZmPIP2.4	--
ZmPIP2.5	--
ZmPIP2.6	--
ZmPIP2.7	--
PpPIP2.1	--
PpPIP2.2	--
PpPIP2.3	--
PpPIP2.4	--
PpPIP3.1	--
AtTIP1.1	--
AtTIP1.2	--
AtTIP1.3	--
AtTIP2.1	--
AtTIP2.2	--
AtTIP2.3	--
AtTIP3.1	--
AtTIP3.2	--
AtTIP4.1	--
AtTIP5.1	--
ZmTIP1.1	--
ZmTIP1.2	--
ZmTIP2.1	--
ZmTIP2.2	--
ZmTIP2.3	--
ZmTIP3.1	--
ZmTIP3.2	--
ZmTIP4.1	--
ZmTIP4.2	--
ZmTIP4.3	--
ZmTIP4.4	--
ZmTIP5.1	--
PpTIP6.1	--
PpTIP6.2	--
PpTIP6.3	--
PpTIP6.4	--
AtNIP1.1	--
AtNIP1.2	R---
AtNIP2.1	--
AtNIP3.1	SQDEICVLRVVDPANQNYFICSSPTDINGKCNVTCKLA------------------
AtNIP4.1	SSSKT---
AtNIP4.2	SKSDG---
AtNIP5.1	--
AtNIP6.1	--
AtNIP7.1	--
ZmNIP1.1	ASA---
ZmNIP2.1	S-VAVDDDELDHIQV---
ZmNIP2.2	--LAADEFDTV---
ZmNIP2.3	SALAADEFDTV---
ZmNIP3.1	--
PpNIP5.1	--
PpNIP5.2	--
PpNIP5.3	--
PpNIP3.1	--
PpNIP6.1	--
AtSIP1.2	--
AtSIP1.1	--
AtSIP2.1	--
ZmSIP1.1	--
ZmSIP1.2	--
ZmSIP2.1	--
PpSIP1.1	EGQAAKSKGLKKESTGNAGDKMKAS-------------------------------
PpSIP1.2	EGESAKDKKRGEGLSENAAGKVKAS-------------------------------
PpGIP1.1	--
PpHIP1.1	--
PpXIP1.1	LACGNEKDGERIRSDSSEDQSSSHHRGKPLTYGRDHAV------------------
PpXIP1.2	GFGPRCPTMFYPSIDQEVDHSQQLVSGNIPTMMSSELLQQPSNQLNSAANKINLVL

AtPIP1.1	- -
AtPIP1.2	- -
AtPIP1.3	- -
AtPIP1.4	- -
AtPIP1.5	- -
ZmPIP1.1	- -
ZmPIP1.2	- -
ZmPIP1.3	- -
ZmPIP1.4	- -
ZmPIP1.5	- -
ZmPIP1.6	- -
PpPIP1.1	- -
PpPIP1.2	- -
PpPIP1.3	- -
AtPIP2.1	- -
AtPIP2.2	- -
AtPIP2.3	- -
AtPIP2.4	- -
AtPIP2.5	- -
AtPIP2.6	- -
AtPIP2.7	- -
AtPIP2.8	- -
ZmPIP2.1	- -
ZmPIP2.2	- -
ZmPIP2.3	- -
ZmPIP2.4	- -
ZmPIP2.5	- -
ZmPIP2.6	- -
ZmPIP2.7	- -
PpPIP2.1	- -
PpPIP2.2	- -
PpPIP2.3	- -
PpPIP2.4	- -
PpPIP3.1	- -
AtTIP1.1	- -
AtTIP1.2	- -
AtTIP1.3	- -
AtTIP2.1	- -
AtTIP2.2	- -
AtTIP2.3	- -
AtTIP3.1	- -
AtTIP3.2	- -
AtTIP4.1	- -
AtTIP5.1	- -
ZmTIP1.1	- -
ZmTIP1.2	- -
ZmTIP2.1	- -
ZmTIP2.2	- -
ZmTIP2.3	- -
ZmTIP3.1	- -
ZmTIP3.2	- -
ZmTIP4.1	- -
ZmTIP4.2	- -
ZmTIP4.3	- -
ZmTIP4.4	- -
ZmTIP5.1	- -
PpTIP6.1	- -
PpTIP6.2	- -
PpTIP6.3	- -
PpTIP6.4	- -
AtNIP1.1	- -
AtNIP1.2	- -
AtNIP2.1	- -
AtNIP3.1	- -
AtNIP4.1	- -
AtNIP4.2	- -
AtNIP5.1	- -
AtNIP6.1	- -
AtNIP7.1	- -
ZmNIP1.1	- -
ZmNIP2.1	- -
ZmNIP2.2	- -
ZmNIP2.3	- -
ZmNIP3.1	- -
PpNIP5.1	- -
PpNIP5.2	- -
PpNIP5.3	- -
PpNIP3.1	- -
PpNIP6.1	- -
AtSIP1.2	- -
AtSIP1.1	- -
AtSIP2.1	- -
ZmSIP1.1	- -
ZmSIP1.2	- -
ZmSIP2.1	- -
PpSIP1.1	- -
PpSIP1.2	- -
PpGIP1.1	- -
PpHIP1.1	- -
PpXIP1.1	- -
PpXIP1.2	Q V K A F M Q P R T G E T R Q A S K T N I M D G A R I D Q N L L L Q Q Y S N S L G S S D G I A I H H S G D F E K

AtPIP1.1	-	-
AtPIP1.2	-	-
AtPIP1.3	-	-
AtPIP1.4	-	-
AtPIP1.5	-	-
ZmPIP1.1	-	-
ZmPIP1.2	-	-
ZmPIP1.3	-	-
ZmPIP1.4	-	-
ZmPIP1.5	-	-
ZmPIP1.6	-	-
PpPIP1.1	-	-
PpPIP1.2	-	-
PpPIP1.3	-	-
AtPIP2.1	-	-
AtPIP2.2	-	-
AtPIP2.3	-	-
AtPIP2.4	-	-
AtPIP2.5	-	-
AtPIP2.6	-	-
AtPIP2.7	-	-
AtPIP2.8	-	-
ZmPIP2.1	-	-
ZmPIP2.2	-	-
ZmPIP2.3	-	-
ZmPIP2.4	-	-
ZmPIP2.5	-	-
ZmPIP2.6	-	-
ZmPIP2.7	-	-
PpPIP2.1	-	-
PpPIP2.2	-	-
PpPIP2.3	-	-
PpPIP2.4	-	-
PpPIP3.1	-	-
AtTIP1.1	-	-
AtTIP1.2	-	-
AtTIP1.3	-	-
AtTIP2.1	-	-
AtTIP2.2	-	-
AtTIP2.3	-	-
AtTIP3.1	-	-
AtTIP3.2	-	-
AtTIP4.1	-	-
AtTIP5.1	-	-
ZmTIP1.1	-	-
ZmTIP1.2	-	-
ZmTIP2.1	-	-
ZmTIP2.2	-	-
ZmTIP2.3	-	-
ZmTIP3.1	-	-
ZmTIP3.2	-	-
ZmTIP4.1	-	-
ZmTIP4.2	-	-
ZmTIP4.3	-	-
ZmTIP4.4	-	-
ZmTIP5.1	-	-
PpTIP6.1	-	-
PpTIP6.2	-	-
PpTIP6.3	-	-
PpTIP6.4	-	-
AtNIP1.1	-	-
AtNIP1.2	-	-
AtNIP2.1	-	-
AtNIP3.1	-	-
AtNIP4.1	-	-
AtNIP4.2	-	-
AtNIP5.1	-	-
AtNIP6.1	-	-
AtNIP7.1	-	-
ZmNIP1.1	-	-
ZmNIP2.1	-	-
ZmNIP2.2	-	-
ZmNIP2.3	-	-
ZmNIP3.1	-	-
PpNIP5.1	-	-
PpNIP5.2	-	-
PpNIP5.3	-	-
PpNIP3.1	-	-
PpNIP6.1	-	-
AtSIP1.2	-	-
AtSIP1.1	-	-
AtSIP2.1	-	-
ZmSIP1.1	-	-
ZmSIP1.2	-	-
ZmSIP2.1	-	-
PpSIP1.1	-	-
PpSIP1.2	-	-
PpGIP1.1	-	-
PpHIP1.1	-	-
PpXIP1.1	-	-
PpXIP1.2	D	R
